# Key Stakeholders' Knowledge, Opinions, and Interests on Real‐World Evidence in the Regulatory Process—Results of an EU‐Wide Survey

**DOI:** 10.1111/cts.70454

**Published:** 2025-12-20

**Authors:** Frank Lucas Depner, Martin Russek, Christoph Röthlein, Cornelia Becker, Jonas Peltner, Kerstin Pfeifer, Evy Reviers, Dirk De Valck, Julia Wicherski, Sirpa Hartikainen, Anna‐Maija Tolppanen, Britta Haenisch

**Affiliations:** ^1^ German Center for Neurodegenerative Diseases (DZNE) Pharmacoepidemiology in Neurodegenerative Disorders Bonn Germany; ^2^ Federal Institute for Drugs and Medical Devices (BfArM) Research Division, Pharmacoepidemiology Bonn Germany; ^3^ European Organisation for Professionals and People with ALS (EUpALS) Leuven Belgium; ^4^ School of Pharmacy University of Eastern Finland Kuopio Finland; ^5^ Center for Translational Medicine University of Bonn Bonn Germany

**Keywords:** patients, RWD, RWE, stakeholders, survey

## Abstract

Real‐world data (RWD) and real‐world evidence (RWE) are increasingly gaining attention in supporting drug regulatory decision making. This study assessed knowledge, opinions, and usage patterns of key stakeholders regarding the status of RWD/RWE and AI implementation in health technology assessment (HTA) and drug regulation and aimed to identify the primary obstacles hindering adoption of these technologies. Four surveys tailored to different stakeholders were created and disseminated online to the respective target groups, including I) regulatory authorities, HTA bodies, and the pharmaceutical industry, II) academia, III) payers, and IV) patients and physicians. The responses were analyzed using descriptive statistics or qualitative content analysis with inductive coding. The survey was active from May 6, 2024 to June 30, 2024. Altogether, 221 respondents participated in the survey. Among respondents from regulatory/HTA authorities or industry, 75.4% viewed RWD/RWE as important for future decision making. Respondents from this group already using RWD (*n* = 56) most frequently reported obstacles regarding data quality (89.3%), data access (62.5%), and data‐coding standardization (57.1%). Patients and physicians predominantly had positive expectations about the use of RWD/RWE, and 94.3% indicated willingness to share healthcare data for research, but all respondents also expressed concerns, with data privacy being most frequently mentioned (75.5%). The results show that although stakeholders are optimistic about RWD/RWE implementation into regulatory practice, our survey suggests that successful implementation may benefit from further development in several areas, including guideline harmonization, RWD infrastructure optimization and accessibility, and professional education.

## Study Highlights

- What is the current knowledge on the topic?
  ○ Real‐world evidence has been gaining attention over the past year, yet different stakeholders have different opinions on its value in drug regulation and health technology assessment.
- What question did this study address?
  ○ What knowledge and opinions do stakeholders have regarding real‐world evidence and artificial intelligence? How do they currently use real‐world data and artificial intelligence?
- What does this study add to our knowledge?
  ○ Real‐world evidence is expected to be important in regulatory decision making in the future by all stakeholder groups. Gaps in guidelines concerning a range of topics in RWD and AI use could be identified. Patients and physicians are willing to share their healthcare data in various contexts, but have some concerns.
- How might this change clinical pharmacology or translational science?
  ○ While our study shows that all stakeholders in the regulatory and health technology assessment process are open about real‐world data usage, several challenges should be addressed in the future, such as improved data access, data quality, and transparency.

## 1 Introduction

Randomized controlled trials (RCTs) continue to be the gold standard for evidence generation in drug regulatory affairs (DRA), including health technology assessment (HTA). RCTs are valued for their high internal validity and ability to establish causal relationships between drug exposure and outcomes [1]. However, RCTs have limitations in their external validity, particularly for certain patient groups often excluded, like children, older adults, pregnant people, or those with chronic comorbidities, which represent a large percentage of patients [2, 3, 4, 5, 6]. Additionally, RCTs face challenges such as cost, duration, and patient recruitment [7]. Real‐world data (RWD) is defined by the European Medicines Agency (EMA) as “routinely collected data relating to patient health status or the delivery of health care from a variety of sources other than traditional trials” [8] and real‐world evidence (RWE) as “information derived from analysis of RWD.” [9] Primary sources of RWD include electronic health records, registry data, claims data, and patient‐reported outcomes, with each offering different information and insights. RWE can complement RCTs within the regulatory process to obtain information which RCTs are unable to measure. Examples include, but are not limited to, the study of populations underrepresented in clinical trials, the study of rare diseases with limited patient pools for RCT recruitment, identification of rare or long‐term side effects of drugs, and the assessment of real‐world effectiveness in representative populations [2].

In Europe, the strategy paper “Regulatory Science to 2025” published in 2020 represents a roadmap for the implementation of RWD/RWE into regulatory practice [10]. Together with the Heads of Medicines Agency (HMA), EMA established the “Joint Task Force on Big Data,” which is responsible for developing methods, approaches, and solutions to allow the use of big data and RWD in EU medicines regulations [11]. RWD infrastructure projects such as the European Health Data Space (EHDS) [12] and the Data Analysis and Real World Interrogation Network (DARWIN EU) [13] are ongoing, and first guidelines on the use of RWD/RWE have been published [14, 15, 16, 17, 18]. However, recent studies have shown that so far, RWD/RWE is mostly used in the post‐marketing stage of the drug lifecycle [19]. In feedback on studies employing RWD/RWE, regulatory authorities frequently cite concerns, including methodological flaws, issues with sample size, missing data, and overall lack of persuasiveness [20].

This study is part of the Horizon EU‐funded research project Real4Reg, which aims to enable the use of RWD/RWE in regulatory decision making by developing standards, methodologies, and tools for the analysis of RWD [21]. To ensure that the project's outputs meet the needs of key stakeholders, this study aims to gather comprehensive information on the knowledge, current usage, and opinions of key stakeholders regarding the status of RWD/RWE implementation in DRA with a focus on the EU. The results are expected to help understand major obstacles and the requirements of stakeholders, as well as to identify gaps in knowledge about these emerging technologies to effectively steer efforts for workforce education.

## 2 Methods

### 2.1 Study Design and Survey Development

A multi‐stakeholder online survey study was conducted to explore the stakeholders' knowledge, opinions, and current usage patterns, including challenges encountered regarding RWD/RWE implementation in DRA.

**FIGURE 1:**
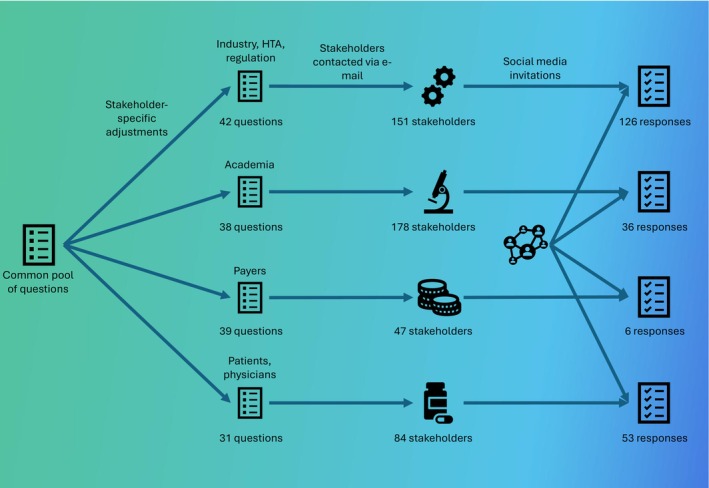
Survey design. HTA Health Technology Assessment.

A summary of the survey setup is shown in Figure 1. To appropriately address different stakeholder activities and involvement in DRA, four distinct target groups of stakeholders were pre‐defined, each receiving an adapted version of the survey. Group one (survey I) comprised the regulatory and HTA community and the pharmaceutical industry—36 drug regulatory agencies, 21 HTA agencies, 39 pharmaceutical companies, and 55 additional entities such as pharmaceutical industry associations, contract research organizations (CROs), and health technology companies were contacted. Group two (survey II) targeted academia, encompassing 143 university departments from relevant fields, 12 academic societies, and 23 research consortia. Group three (survey III) consisted of 47 European health insurance companies and providers and their associations. Group four (survey IV) focused on patients and physicians, reached through 49 medical and patient associations and 35 healthcare professional associations.

Based on existing literature and the two previous surveys conducted by the HMA/EMA Joint Task Force on Big Data, “Survey for National Competent Authorities” [22] and “Survey for Pharmaceutical Industry,” [23] dimensions for this survey were derived. The “EMA Training Curriculum on Big Data for regulators” [24] was consulted to inform questions related to the state of knowledge of stakeholders on relevant RWD‐/RWE‐related topics. Furthermore, experts from relevant areas within the Real4Reg project were consulted to refine each item of the four surveys.

The number of questions varied between 31 and 42 items, with many questions overlapping between the surveys, taking approximately 15 min to answer. All items could be skipped; skipped questions are reported as ‘No answer’ in the results. Responses were only recorded when participants confirmed submission of the results on the last page of the survey. Surveys I–III were divided into the categories “General Information” (covering demographic aspects), “Knowledge,” “Opinions,” “Current usage,” and “Interest.” Survey IV was structured identically, with the omission of the “Current usage” section, and increased focus on exploring participants’ awareness of the topic and their opinions on various aspects of data sharing. The questionnaires are included in Supplement 1.

The survey was conducted using the platform EUSurvey (https://ec.europa.eu/eusurvey) between May 6, 2024 and June 30, 2024. A link to the survey was included in each email invitation to the specific stakeholders identified. To increase the response rate, two reminders were sent out at intervals of 3 weeks. Additional dissemination took place via social media (LinkedIn) and the Real4Reg project's newsletter.

### 2.2 Analysis

The analysis of quantitative data was descriptive. For single‐choice and multiple‐choice questions, the relative frequency of each answering option selected was calculated. For the matrix questions, an average score was calculated for each skill item, based on the response options on the Likert scale (0–5). A “skill gap” was then calculated by subtracting the average of respondents' assessments for the future importance of each skill item from the average of respondents' self‐rated knowledge levels. For survey I, a subgroup analysis was conducted stratified based on the type of employer. The matrix questions and derived skill gap were also stratified depending on whether respondents indicated already using RWD/RWE or AI‐based algorithms. For survey IV, a subgroup analysis was conducted based on the respondents' role as either patient or physician.

For qualitative analysis of the closing free‐text question, content analysis with conventional (inductive) coding was used [25]. Each response could be assigned to multiple first‐level codes but could only count once toward each individual first‐level code. The results of the qualitative analysis are reported in Supplement 3.

All analyses were performed in R version 4.4.

## 3 Results

### 3.1 Surveys I–III

#### 3.1.1 Demographics

After contacting 151 stakeholders directly, a total of 126 participants took part in survey I, of which 67% originated from Germany and 33% from 15 other European countries (Supplement 2 Table S1). For survey II, 178 direct survey invitations led to 36 responses, with 25% from Germany and the remainder spread over 13 European countries and the USA (Supplement 2 Table S2). Survey III only received six responses and was thus not analyzed to preserve the privacy of respondents.

Among survey I respondents, 91 (72.2%) indicated working for a drug regulatory agency, 9 (7.1%) at HTA agencies, and 24 (19.1%) in the pharmaceutical industry. Two respondents (1.6%) did not specify. The primary work domains reported were regulatory and compliance (*n* = 57, 45.2%), data analytics (*n* = 32, 25.4%), and medical and scientific affairs (*n* = 16, 12.7%). In survey II, most of the respondents (*n* = 19, 52.8%) identified (pharmaco‐)epidemiology and public health as their primary area of expertise, followed by biostatistics and data science, pharmacology and toxicology, and clinical research (all *n* = 4, 11.1%).

#### 3.1.2 Knowledge, Future Importance, and Skill Gap

**FIGURE 2:**
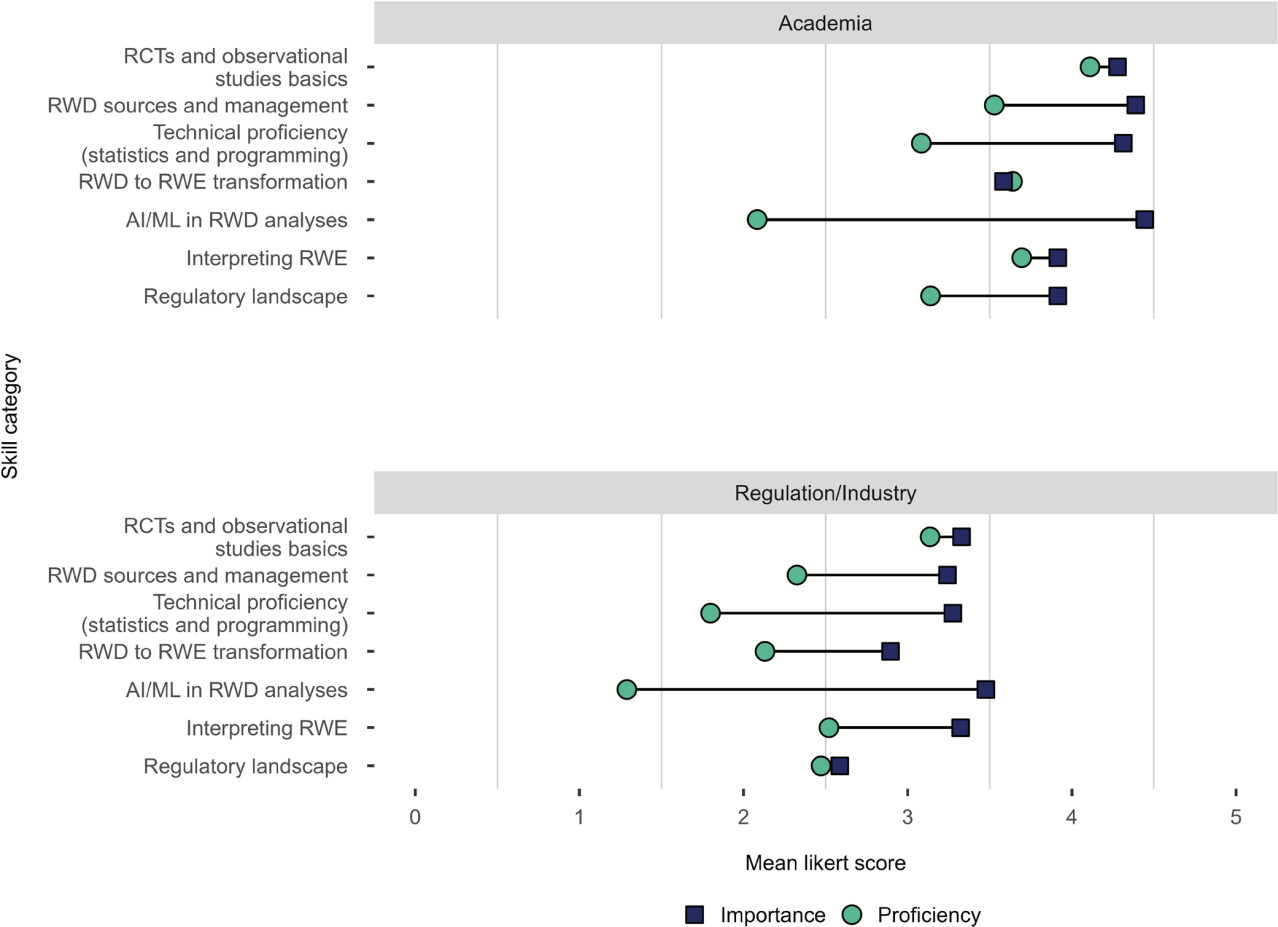
Average Likert scores of self‐rated proficiency and perceived importance of different RWD‐related topics for respondents of survey I (*n* = 126) and survey II (*n* = 36). AI, Artificial Intelligence; ML, Machine Learning; RCT, Randomized Controlled Trial; RWD, Real‐World Data, RWE, Real‐World Evidence.

In the evaluation of respondents' proficiency across different RWD‐/RWE‐related skill items and their assessment of the future importance of proficiency in those topics, nearly all topics received higher ratings for future importance compared to current skill level. Academics rated both their own proficiency as well as the future importance of the skill topics higher than respondents working in regulatory agencies or industry. The largest gaps between perceived importance and skill self‐rating (“skill gaps”) in both target groups were identified in “AI/ML in RWD analyses” and “Technical proficiency (statistics and programming).” For regulatory, HTA, and pharmaceutical industry representatives, skill gaps were also detected in the fields of “RWD sources and management,” “RWD to RWE transformation,” and “Interpreting RWE.” Participants from academic institutions showed a skill gap in “RWD sources and management” and “Regulatory landscape” (Figure 2).

**FIGURE 3:**
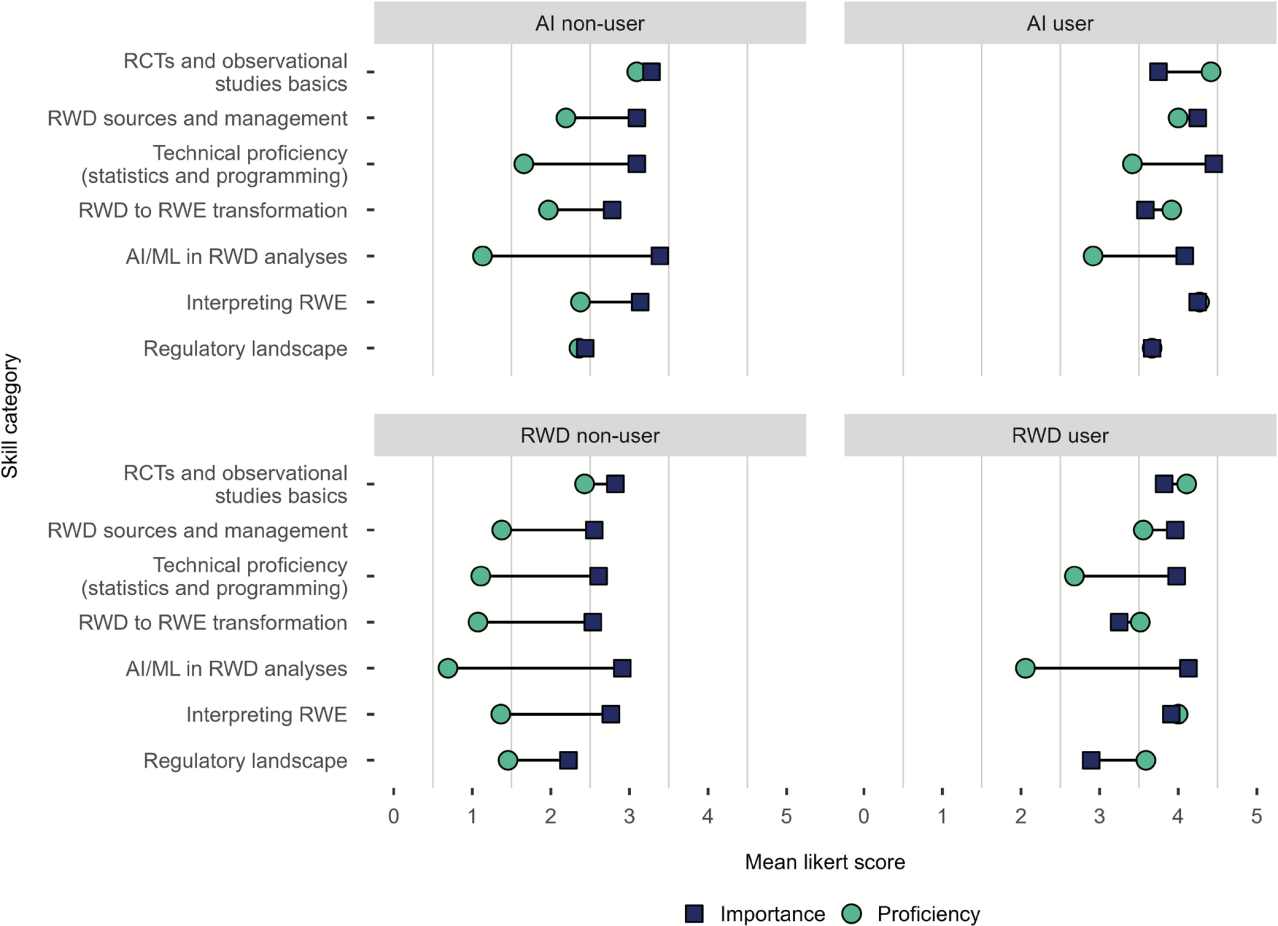
Average Likert scores of self‐rated proficiency and perceived importance of different RWD‐related topics for respondents of survey I (*n* = 126), stratified by AI use and RWD use. 12 respondents for AI user, 96 for AI non‐user; 56 respondents each for RWD user and non‐user. AI, Artificial Intelligence; ML, Machine Learning; RCT, Randomized Controlled Trial; RWD, Real‐World Data, RWE, Real‐World Evidence.

Stratification by employer type in survey I only showed minor differences between groups (Supplement 2 Figure S1). Analysis stratified by respondents reporting current RWD use and those reporting already using AI‐based algorithms demonstrated lower skill gaps compared to respondents not yet using RWD or AI‐based algorithms. Yet, skill gaps in the categories “Technical proficiency (statistics and programming)” and “AI/ML in RWD analysis” were also present for RWD and AI users (Figure 3).

#### 3.1.3 Opinions

Respondents were asked to rate how important they consider the use of RWD/RWE and AI/ML to support regulatory decision making in the future. Both technologies were consistently rated positively, with 75.4% (*n* = 95) of survey I respondents rating RWD/RWE as important or higher, compared to 63.5% (*n* = 80) for AI/ML algorithms. Academic respondents assigned even higher importance to both technologies, with 97.2% (*n* = 35) rating RWD/RWE and 77.8% (*n* = 28) rating AI/ML as important or above. Among respondents of survey I, pharmaceutical industry representatives rated these technologies' future importance highest, followed by regulators, with HTA experts assigning the lowest importance ratings. Further details can be found in Supplement 2 Figures S2–S5.

#### 3.1.4 Current Usage of RWD/RWD and AI

In survey I, equal proportions of respondents (*n* = 56, 44.4%) reported either currently using or not using RWD/RWE for regulatory purposes. Usage rates were higher among pharmaceutical industry respondents (*n* = 20, 83.3%) compared to those from regulatory (*n* = 32, 35.2%) and HTA communities (*n* = 4, 44.4%) (Supplement 2 Figure S6). In survey II, 94.4% (*n* = 34) respondents reported using RWD for research purposes.

**FIGURE 4:**
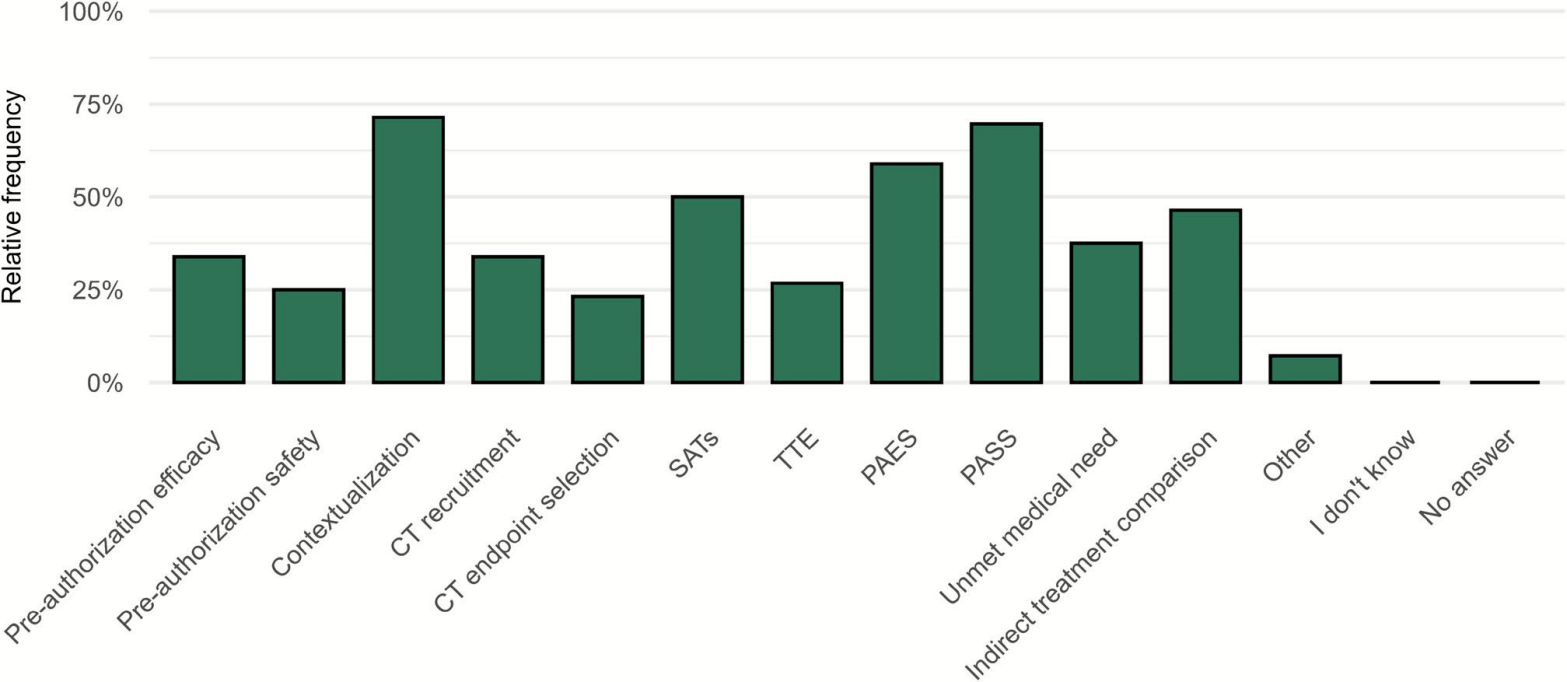
Current use cases of RWD for RWD users in survey I (*n* = 56). More than one response could be selected. CT, Clinical Trial; PAES, Post‐Authorisation Effectiveness Study; PASS, Post‐Authorisation Safety Study; SAT, Single‐Arm Trial; TTE, Target Trial Emulation.

Figure 4 shows the usage categories of RWD among RWD users in survey I. The most frequently reported applications were (disease) contextualization (*n* = 40, 71.1%), post‐authorization safety analysis (*n* = 39, 69.6%), and post‐authorization efficacy analysis (*n* = 33, 58.9%). Applications with the lowest reported use were pre‐authorization efficacy assessments (*n* = 19, 33.9%), pre‐authorization safety assessments (*n* = 14, 25.0%), and applications in clinical trial design such as recruitment (*n* = 19, 33.9%) and endpoint selection (*n* = 13, 23.2%). Pharmaceutical industry respondents additionally reported high utilization rates for evaluating unmet medical needs, for indirect treatment comparisons, and as external control arms (all *n* = 14, 70.0%) (Supplement 2 Figure S7).

**FIGURE 5:**
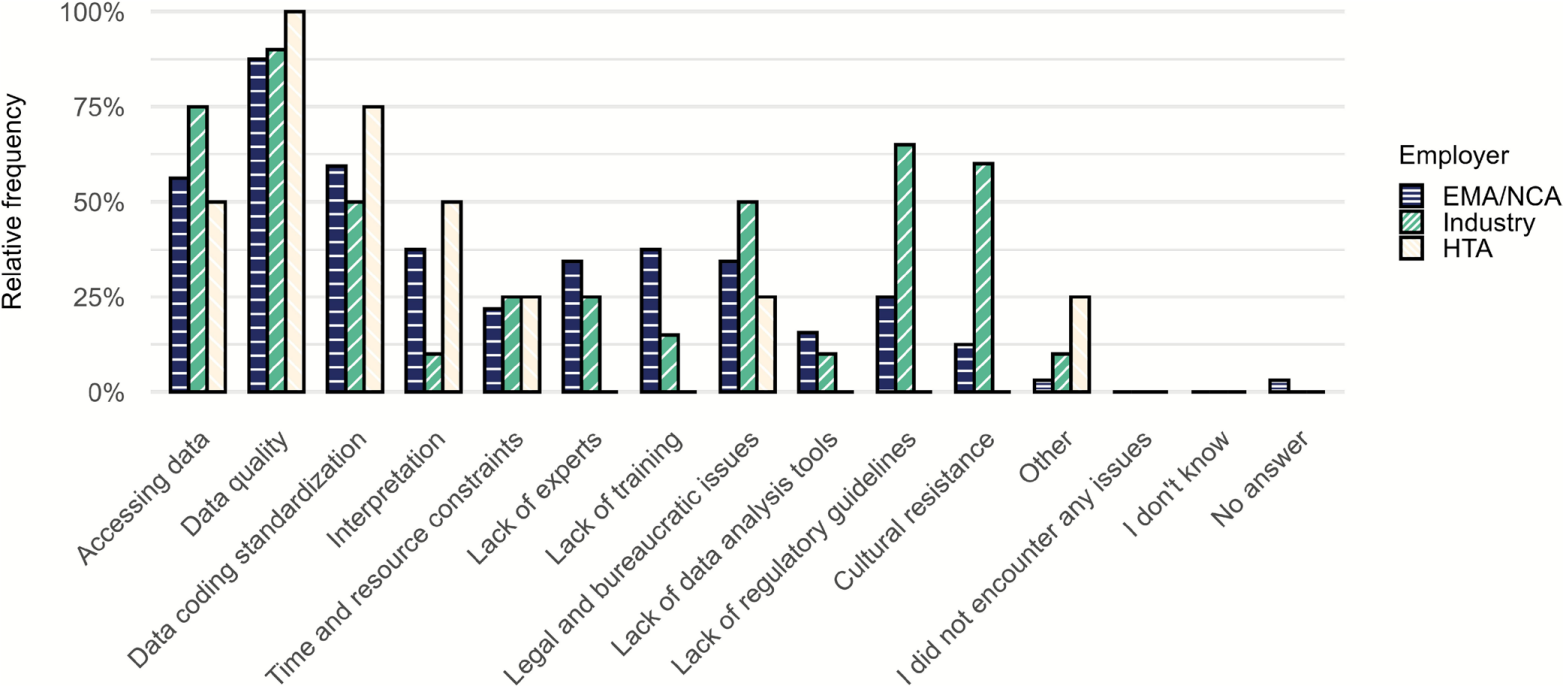
Challenges encountered when making use of RWD/RWE for regulatory purposes by RWD users in survey I, stratified by employer type. Note that employer types Consulting, CRO and Health technology have been included in the Industry block. More than one response could be selected. 32 respondents from NCA/EMA, 20 from Industry, 4 from HTA. EMA, European Medicines Agency; HTA, Health Technology Assessment; NCA, National Competent Authority.

Among participants of survey I who already use RWD/RWE in DRA, the most prevalent challenges identified were data quality (*n* = 50, 89.2%), data access (*n* = 35, 62.5%), and data coding standardization (*n* = 32, 57.1%). Industry representatives further highlighted regulatory guideline availability (*n* = 13, 65.0%) and cultural resistance to RWD/RWE use (*n* = 12, 60.0%) (Figure 5). Most non‐users of RWD/RWE (*n* = 28, 50.0%) indicated that they did not encounter the need for RWD/RWE as the reason for not using RWD/RWE in DRA applications. Other barriers were data quality, cultural resistance (both *n* = 12, 21.4%), and data access (*n* = 11, 19.6%). Academics reported similar challenges regarding RWD/RWE use for application in research (Supplement 2 Figure S8).

In survey I, 12 respondents (9.5%) reported already making use of AI‐based algorithms for the analysis of RWD related to DRA. Most frequently reported use cases were the analysis of patient subsets (*n* = 7, 58.3%) and the generation of synthetic data (*n* = 6, 50.0%) (Supplement 2 Figure S9). Within the academic group, 11 respondents (30.6%) reported current use of AI‐based algorithms for RWD analysis. Applications spanned a broad range, including data structuring, signal detection, clinical text analysis, imaging information processing, and predictive modeling.

In survey I, distinct patterns of challenges emerged between AI‐users and non‐users regarding the implementation of AI‐based algorithms for RWD analysis for DRA‐related purposes. Users primarily reported challenges with training data availability (50.0%), model generalization (41.7%), model validation (41.7%), and explainable AI (33.3%). Non‐users cited lack of applications requiring AI‐based algorithms (39.6%), limited model access (33.3%), and regulatory constraints (21.9%) as primary barriers. Insufficient availability of skilled personnel was reported by both AI‐users (41.7%) and non‐users (19.8%) (Table 1).

**TABLE 1:** Obstacles reported by AI users (*n* = 12) and AI non‐users (*n* = 96) in survey I. Respondents could select more than one response.

Challenge	AI users	Non‐AI users
No access to AI/ML models at all	1 (8.3%)	32 (33.3%)
Model validation issues	5 (41.7%)	8 (8.3%)
Model generalization and overfitting issues	5 (41.7%)	4 (4.2%)
Lack of (relevant) training datasets	6 (50.0%)	15 (15.6%)
Interpretability issues/explainable AI issues	4 (33.3%)	11 (11.5%)
Reproducibility issues	2 (16.7%)	5 (5.2%)
Lack of computing power	3 (25.0%)	4 (4.2%)
Integration issues (problems integrating the new models into existing IT systems or workflows)	1 (8.3%)	13 (13.5%)
Regulatory and compliance challenges (e.g., due to unclear regulatory guidelines or data privacy issues)	2 (16.7%)	21 (21.9%)
Acceptance and trust issues toward AI/ML models by other stakeholders	3 (25.0%)	12 (12.5%)
Lack of skilled personnel	5 (41.7%)	19 (19.8%)
Other	1 (8.3%)	8 (8.3%)
I currently don't require AI‐/ML‐based algorithms	0 (0.0%)	38 (39.6%)
I don't know	0 (0.0%)	9 (9.4%)
No answer	1 (8.3%)	3 (3.1%)
Total respondents	12	96

Abbreviations: AI, Artificial Intelligence; IT, Information Technology; ML, Machine Learning.

The use of 11 different common data models (CDMs) was reported across all survey stakeholder groups, with the Observational Medical Outcomes Partnership (OMOP) CDM [26] reported as the most frequently utilized CDM (Supplement 2 Figures S10 and S11).

#### 3.1.5 Guideline Use

Among stakeholders assessed in survey I, 49 (38.9%) reported using (draft) guidelines, frameworks, or similar guidance documents about the use of RWD/RWE for DRA. Usage rates were higher among pharmaceutical industry respondents (*n* = 24, 62.5%) compared to HTA respondents (*n* = 9, 55.6%) and drug regulators (*n* = 91, 31.9%).

Survey respondents reported several limitations of current guidelines. These concerned data quality standards, integration of multiple RWD sources, the use of AI‐based algorithms (all *n* = 29, 46.9%), study submission guidance (*n* = 16, 32.7%), and EU‐wide (*n* = 19, 38.8%) as well as global guideline harmonization (*n* = 17, 34.7%) (Supplement 2 Figure S12).

### 3.2 Survey IV

#### 3.2.1 Demographics

In survey IV, after directly inviting 84 stakeholders from the groups of patients and physicians to participate, 53 responses were collected. A large share of respondents (47.0%) was from Portugal, with the remainder being distributed across more than 18 EU and non‐EU countries (Supplement 2: Table S3). Among respondents, 27 (50.9%) completed the survey as patients, 23 (43.5%) as physicians, and 3 (5.7%) did not specify their role.

#### 3.2.2 Knowledge

Within the sample, 71.7% (*n* = 38) of respondents reported having heard about RWD/RWE in the context of healthcare data before, with a higher proportion among physicians (*n* = 18, 78.2%) than among patients (*n* = 18, 66.7%). Most patients (*n* = 17, 63%) report that they were never educated on the topic of RWD usage for medical research. When physicians were asked whether they felt confident of educating their patients on the topic of RWD/RWE for medical research purposes, 60.8% (*n* = 14) felt completely or somewhat comfortable in doing so, while 39.1% (*n* = 9) felt not very confident.

#### 3.2.3 Opinions

Most respondents (*n* = 48, 91.0%) estimated the future impact of RWD/RWE for patient care as “important” or higher. The importance to educate patients and physicians about the topic of RWD/RWE was also rated as “important” or higher by 86.8% (*n* = 48) of respondents.

**FIGURE 6:**
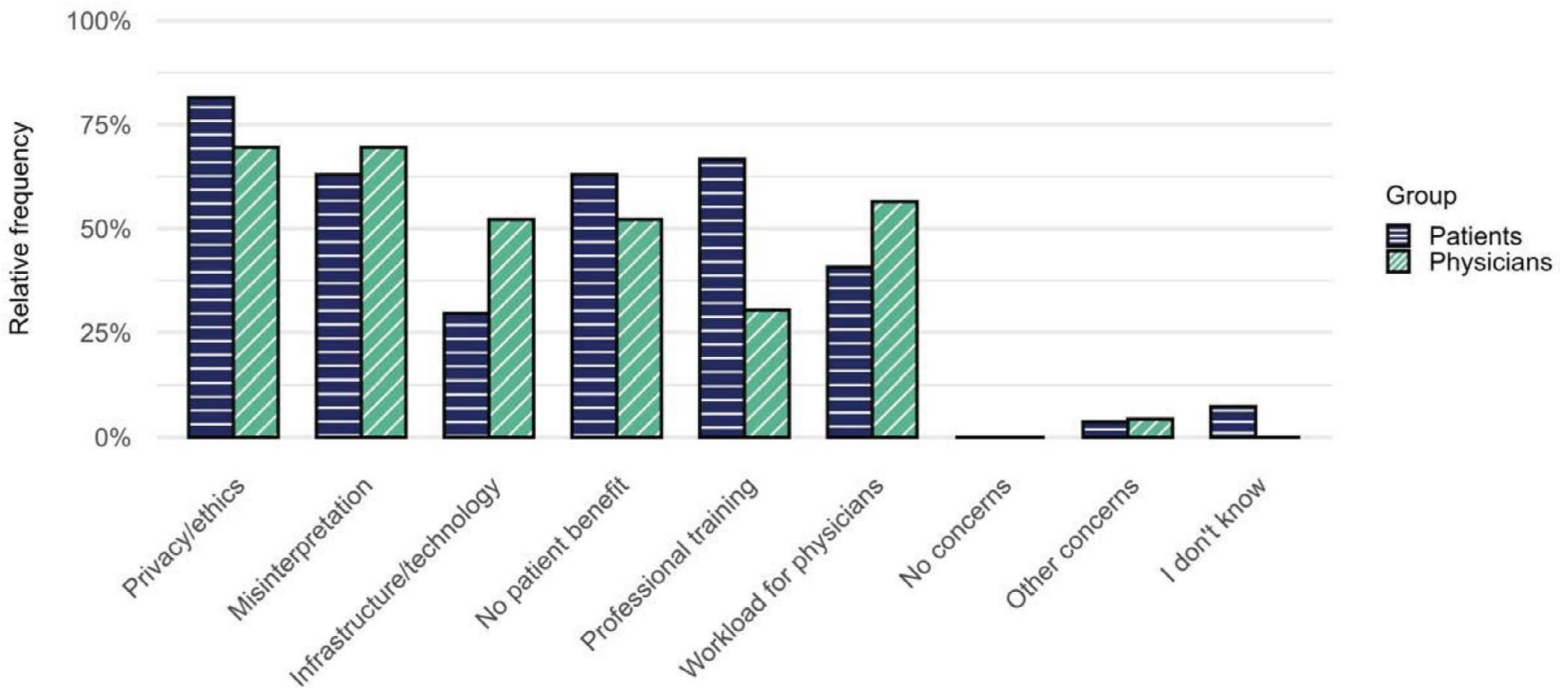
Main concerns reported by patients (*n* = 27) and physicians (*n* = 23) regarding the use of RWD/RWE for clinical research purposes. More than one response could be selected.

Overall, respondents indicated a high willingness to share healthcare data with researchers. Specifically, patients and physicians reported openness to data sharing for academic research purposes (*n* = 25, 92.6% and *n* = 19, 82.6%, respectively), public health research and monitoring (*n* = 26, 96.3% and *n* = 16, 69.6%), and with regulatory and other government agencies (*n* = 24, 88.9% and *n* = 14, 60.1%). The lowest willingness to share healthcare data was assigned to private sector research (*n* = 16, 59.3% and *n* = 6, 26.1%) (Supplement 2 Figure S13). At the same time, most respondents also voiced concerns about the use of RWD/RWE. Data privacy and ethics emerged as the predominant concern (*n* = 22, 81.1% and *n* = 16, 69.6%), followed by misinterpretation of RWD‐derived insights (*n* = 17, 63.0% and *n* = 16, 70.0%), and potential for a lack of benefits for patients (*n* = 17, 63.0% and *n* = 12, 52.2%) (Figure 6).

## 4 Discussion

### 4.1 Views of Stakeholders Toward the Implementation of RWD/RWE Into DRA

This study examined RWD/RWE implementation across key stakeholders in the EU, revealing a gap between future expectations and current practice. While stakeholders view RWD/RWE as promising and increasingly important for regulatory decisions, present applications remain largely confined to established domains such as post‐authorization studies and academic pharmacoepidemiological research. Usage patterns varied between different stakeholders, with higher adoption rates in academia and the pharmaceutical industry compared to regulatory authorities. The use of AI‐based algorithms shows potential in various medical applications, including the analysis of RWD [27, 28]. Yet, despite recognized potential across stakeholder groups, current adoption rates remain low.

The challenges hindering RWD/RWE adoption extend beyond workforce education to encompass both technical and systemic barriers. In terms of technical barriers, data quality emerged as the predominant concern. Respondents emphasized the need for fit‐for‐purpose data able to meet the specific research questions arising during the drug regulatory process and report issues such as missing data, insufficient data granularity, and a lack of quality indicators. At the systemic level, data access and regulatory framework issues emerged as primary obstacles. Respondents reported difficulties caused by current data protection legislation which limits access to existing RWD sources. The identified challenges, together with the cultural resistance toward RWD reported in the free text answers, might explain the quarter of respondents not viewing RWD as important in the future.

These findings, both in terms of respondents' opinion on the future importance of RWD/RWE and in terms of current challenges, are largely in line with previous studies investigating different stakeholders. A Deloitte survey of pharmaceutical industry executives reported similar expectations about RWD/RWE's future importance, though with higher current usage rates than our study [29]. Patadia et al. [30] confirmed widespread RWD use in pharmacovigilance among pharmaceutical companies—electronic health records and claims databases were also identified as most frequently used RWD sources, but they reported lower use of registries. Compared to the EMA‐HMA survey to industry [31] from 2017, the main challenges of data quality and data access remain constant, while the lack of regulatory guidelines could be identified as a new challenge in our survey.

Our findings are in line with findings from different previous studies regarding key challenges, particularly around access to RWD sources being hampered by data privacy regulations, low cooperation between stakeholders, and a lack of validation, quality indicators, and standardization of RWD sources [30, 32, 33, 34]. Fewer studies have investigated the views of regulators or HTA organizations. In a survey among EUnetHTA member organizations, Hogervorst et al. [35] also found that stakeholders indicate the need for wider systematic use of RWD in HTA decisions, but report data access, harmonization with existing policy structures, and issues around data quality and RWE interpretation as major hurdles. Compared to the EMA‐HMA survey to NCAs from 2017, data quality was consistently rated as the biggest challenge, while the lack of expertise was considered a far higher challenge in 2017 compared to our results, potentially reflecting increased recruitment of staff with the relevant skills.

### 4.2 The Views of Patients and Physicians

Patients and physicians offered a distinct perspective on RWD/RWE implementation. While respondents demonstrated a strong willingness to share healthcare data for research and regulatory purposes and expressed optimistic expectations, they viewed sharing with private industry more critically. Primary concerns were centered on data privacy, potential misinterpretation of RWD, and uncertainty about patient benefits. Respondents also voiced the need for better education. The importance of education for these stakeholder groups was highlighted by respondents in our survey showing that a large percentage of physicians only feel somewhat comfortable in educating their patients. These perspectives align with previous research on public attitudes toward health data sharing. Luchenski et al. [36] found high support among UK patients for using electronic healthcare records in healthcare planning and research, provided personal identifiers were removed. Similarly, the PatientsLikeMe study of over 3000 patients by O'Brien et al. [37] revealed widespread willingness to share health data, while emphasizing that privacy protection, transparency, and education about data usage could increase participation. Recent reviews have confirmed these patterns, highlighting concerns about privacy breaches, data misuse, and commercial applications [38, 39]. Research on physician perspectives, though more limited, indicates generally positive attitudes toward RWD/RWE, tempered by concerns about methodological challenges in data interpretation [40].

### 4.3 Recommendations and Current Development

These findings suggest that to address the challenges affecting the implementation of RWD/RWE into DRA practice, future efforts should focus on the standardization of RWD sources and analysis pipelines as well as the harmonization and closing of gaps in regulatory guidelines. While a variety of related guidelines have already been published [14, 15, 16, 17, 18], respondents indicate that the current landscape remains unconsolidated with significant gaps. Progress in this area has been made through the recent publication of an International Council on Harmonisation of Technical Requirements for Registration of Pharmaceuticals for Human Use (ICH) reflection paper on opportunities for harmonization of RWE generation and updates to the ICH M14 guideline on general principles on plan, design, and analysis of pharmacoepidemiological studies that utilize RWD for safety assessment of medicines [41, 42]. In Europe, health data ecosystems such as the EHDS and DARWIN EU could help to address issues such as RWD access and RWD source data quality validation. The EU AI Act and the AI work plan to guide use of AI in medicines regulation represent first initiatives to guide the incorporation of AI technology into DRA [43, 44].

The present findings further suggest that for all stakeholders, educational initiatives and increased cooperation could facilitate the adoption of RWD/RWE and AI by eliminating knowledge gaps and strengthening the interaction between different stakeholders.

Patients and physicians were willing to share their healthcare data, especially for academic and public health research. Building on this generally positive attitude and strengthening communication to citizens about the concerns regarding privacy and misinterpretation will ensure that future RWD research, for example, through the EHDS, guarantees evidence representative of a broad population.

### 4.4 Limitations

While providing a broad overview and insights into RWD/RWE implementation status and stakeholder perspectives, the present study has limitations. Representation varied across stakeholder groups and geographic regions, with lower participation from HTA, pharmaceutical industry representatives, and payers, the latter showing insufficient for analysis. While a response rate cannot be calculated due to invitations also having been shared via social media, it is likely that many invited individuals did not participate in the survey.

For the interpretation of the gaps between self‐reported knowledge and perceived importance of RWD‐related topics (“skill gaps”), the common challenges of self‐assessment need to be acknowledged. At the same time, several studies report high correlation between self‐rated skill levels and those measured through examinations [45, 46, 47]. The relevance of individual skill gaps also differs between different stakeholder groups—while knowledge of AI methodology will be important to individuals using AI, it will be less relevant for those that do not plan to use AI in the future. Therefore, the skill gaps displayed in our survey should be interpreted with the relevant stakeholders' professional context in mind.

Our study contributes important knowledge on skills, opinions on, and usage of RWD through a comprehensive evaluation across stakeholder groups. By using similar but stakeholder‐specific questionnaires, comparison between surveys was possible while still asking questions relevant to each stakeholder group. To our knowledge, this is the first study contrasting stakeholders' skills and perceived importance of different RWD‐ and AI‐related topics, thereby helping to identify specific educational aims for different stakeholder groups.

## 5 Conclusions

Stakeholders widely recognize the growing significance of RWD/RWE in the regulatory process. However, some technical knowledge challenges and a combination of practical and systemic issues currently limit routine implementation. Future efforts to advance RWD/RWE integration into regulatory practice should focus on harmonization of guidelines, data frameworks and analysis pipelines, creation of accessible and validated RWD databases, professional education, and strengthening stakeholder cooperation. Patients and physicians demonstrated overall support for RWD/RWE usage but showed concern regarding data privacy. Accessible training for professionals within the medical community and clear information for patients, physicians, and citizens in general are important to ensure public trust.

## Author Contributions

All authors wrote the manuscript; all authors designed the research; Frank Lucas Depner, Martin Russek, Christoph Röthlein, Cornelia Becker, and Kerstin Pfeifer performed the research; Frank Lucas Depner, Martin Russek and Christoph Röthlein analyzed the data.

## Funding

The research leading to these results has received funding from the European Union’s Horizon Europe Programme under grant agreement no. 101095353 (Real4Reg). The funder did not play a role in study design, execution, or interpretation.

## Conflicts of Interest

The authors declare no conflicts of interest.

## Supporting information


**Data S1:** Supporting Information.
