# Supplementary material for: Key Stakeholders' Knowledge, Opinions, and Interests on Real‐World Evidence in the Regulatory Process—Results of an EU‐Wide Survey
Source: Clin Transl Sci. 2025 Dec 20;18(12):e70454. doi: 10.1111/cts.70454 (PMC12717853; doi:10.1111/cts.70454)
Supplement: Supplementary file 1 — Data S1: Supporting Information. [file CTS-18-e70454-s001.zip › CTS-2025-0743-T-s03.docx]

# Survey I-III

Within the surveys I-III, targeting professional stakeholders directly involved with DRA, 40 responses to the free-text question inquiring about the most pressing challenges hindering adoption of RWD/RWE use in DRA were included in the qualitative analysis. Twelve responses were excluded as they did not address the topic of RWD or AI – those included ‘thank you’ messages and comments on the design of the survey itself.

The analysis revealed two issues in two major categories: technical barriers (32 mentions) and system barriers (48 mentions). In terms of technical barriers, data quality was the most frequently mentioned 1^st^ level code (20 mentions), with respondents mentioning specific issues in terms of a lack of fit-for-purpose data (8 mentions), missing data (5 mentions), problems with data source quality validation (4 mentions), and insufficient data granularity (2 mentions). A lack of suitable analysis methods was mentioned by 12 respondents.

Among system barriers, regulatory and guidance issues were the most frequently reported 1^st^ level code with 17 mentions, with gaps in guidance on RWD collection (6 mentions), analysis and use (6 mentions), and a lack of regulatory harmonization (4 mentions) being mentioned as specific issues. This is followed by obstacles relating to data access, which include data access problems due to data protection legislation (7 mentions) and due to lacking RWD infrastructure (10 mentions). Regarding the latter, respondents specified problems caused by a fragmentation of RWD sources (3 mentions) and limitations on timeliness of data access (3 mentions). Cultural resistance defined as low trust, recognition, and acceptance towards RWD and a lack of incentives to use RWD/RWE was mentioned 10 times. The 1^st^ level codes of lack of education and of cooperation between stakeholders were mentioned 10 and 4 times, respectively. The complete coding table can be seen below:

| **Category (theme)** | **1^st^ level code (topic)** | **2^nd^ level code (specific issue)** |
| --- | --- | --- |
| Technical barriers 32 | Data quality 20 | Missing data 5 |
|  |  | Insufficient granularity 2 |
|  |  | Quality indicators/validation 4 |
|  |  | Fit-for-purpose data 8 |
|  | Suitable analysis methods 12 | Data linkage and integration 4 |
|  |  | Validation of AI/ML algorithms 2 |
|  |  | Standardization of methods 1 |
| System barriers 48 | Regulatory and guidance issues 17 | Guidance on RWD collection 6 |
|  |  | Guidance on RWD analysis and use 6 |
|  |  | Regulatory harmonization 4 |
|  | Data access (Data protection legislation) 7 |  |
|  | RWD infrastructure 10 | Fragmentation of RWD sources 3 |
|  |  | Interoperability of IT systems and platforms 1 |
|  |  | Limitations on timeliness of data access 3 |
|  | Cooperation between stakeholders 4 |  |
|  | Cultural resistance (Trust/recognition/acceptance/incentives) 10 |  |
|  | Education 10 |  |
| Other barriers 14 | Lack of resources 8 |  |
|  | Change management 4 |  |
|  | Ethical concerns 2 | The high quality and standard of RCTs must be kept or patients will lose trust 1 |
|  |  | Data privacy and safety 1 |

Total number of responses: 52

Number of removed responses: 12

Number of responses: 40

# Survey 4

The free-text question inquiring about the main challenges in terms of RWD/RWE implementation into the healthcare system was answered by 13 respondents. The category with the most mentions (13) were technical concerns, which included the 1^st^ level codes of data quality concerns (5 mentions), problems with data availability, collection, and storage (7 mentions), and a lack of suitable analysis methods (1 mention). Concerns about education and awareness was the second most frequently mentioned category (10 mentions), with a lack of education for patients (6 mentions) and physicians (3 mentions) being noted by respondents. This was followed by ethical concerns (9 mentions), such as data privacy issues (3 mentions) or concerns about biased algorithms and data (2 mentions). Practical challenges (5 mentions) reported by respondents include increased workload for physicians (2 mentions) and a lack of cooperation between stakeholders (2 mentions).

The complete coding table can be seen below:

| **Category (theme)** | **1^st^ level code (topic)** |
| --- | --- |
| Technical concerns 13 | Data quality 5 |
|  | Data availability (collection and storage) 7 |
|  | Suitable analysis methods 1 |
| Education and awareness concerns 10 | Education for patients 6 |
|  | Education for physicians 3 |
|  | Education for other parties including the general public 1 |
| Practical concerns 5 | Increased workload for physicians 2 |
|  | Funding for RWD research 1 |
|  | Cooperation between stakeholders 2 |
| Ethical concerns 9 | Fear of increased drug prices 1 |
|  | Data privacy 3 |
|  | Equal access to individualized approaches 1 |
|  | Biased algorithms and data 2 |
|  | Dealing with special (vulnerable) populations 2 |

Total number of responses: 14

Number of removed responses: 1

Number of responses: 13
