# Supplementary material for: Key Stakeholders' Knowledge, Opinions, and Interests on Real‐World Evidence in the Regulatory Process—Results of an EU‐Wide Survey
Source: Clin Transl Sci. 2025 Dec 20;18(12):e70454. doi: 10.1111/cts.70454 (PMC12717853; doi:10.1111/cts.70454)
Supplement: Supplementary file 1 — Data S1: Supporting Information. [file CTS-18-e70454-s001.zip › CTS-2025-0743-T-s01.pdf]

Real4Reg

Real4Reg Survey - Regulatory and Industry

Introduction

Welcome to the Real4Reg Survey on Real-World Data and Real-World Evidence use in Regulatory Affairs and Health Technology Assessment

Background

As defined by the European Medicines Agency (EMA), real-world data (RWD) encompasses “routinely collected data relating to patient health status or the delivery of health care from a variety of sources other than traditional clinical trials.” Real-world evidence (RWE), on the other hand, is “information derived from the analysis of RWD.” While traditionally mainly used for pharmacovigilance purposes, RWE can also be used for pre-authorisation efficacy and safety studies, to supplement and improve the efficacy of clinical trials, and to conduct trials, which would otherwise be very difficult to run. In other words, the use of RWD can increase the safety and efficacy of drugs available to patients, while at the same time improving the drug development process by making it more cost-effective and faster. So far though, RWD and RWE currently only play a limited role in regulatory decision-making and Health technology assessment (HTA). The Real4Reg Project aims to establish their values in these areas.

The Real4Reg Project

Real4Reg is a consortium consisting of ten partners from six different European countries with experience in the field of RWD analyses, including experts from regulatory agencies and HTA bodies (BfArM, DKMA, Infarmed), academia (Fraunhofer, UEF, CSC, AU, DZNE), and patient organisations (EUpALS, EIWH).

To support and facilitate the widespread adoption of RWD/RWE usage in regulatory affairs and HTA, we are developing standards for RWD/RWE use, including artificial intelligence (AI) and machine learning (ML) tools to manage, analyse, and interpret this data. These tools will be validated through a series of good practice examples on currently highly relevant research topics in the field of drug safety, efficacy, and clinical research. We are also creating training modules to fill existing gaps and enhance the RWD/RWE skills within the regulatory and industry sectors. For an in-depth look at our work, visit our website at https://www.real4reg.eu/.

Aim of this survey

This survey is designed to gather a well-rounded view of stakeholders' requirements, knowledge, capacities, and opinions on RWD/RWE. We aim to identify knowledge gaps that could hinder the successful implementation of RWD/RWE in regulatory and HTA processes. Your insights are vital for guiding our training and guidance development.

How to complete the survey

Please only complete this survey if you are currently working for a regulatory agency (National Competent Agency, NCA), an HTA body, or a private company with its primary focus area in the field of pharmaceuticals or medical devices.

Answering the survey will take approximately 10-15 minutes. When answering questions in this survey, please make sure to click the small question mark (?) symbol next to some questions.

By participating in this survey, you are playing a crucial role in our journey. Your expertise and perspective are invaluable assistance to our work.

Thank you for participating!

Data protection

Data collection within this survey

Your data is collected for the purpose of scientifically analyzing the current state of Real-World Data (RWD) and Real-World Evidence (RWE) usage, expertise, and the stakeholder's general opinions towards this topic.

All data collected as part of this survey is anonymized. This means, your responses will not be identifiable to you as an individual. All data gathered during this survey will be aggregated and analyzed. In order to stay anonymous, please do not insert any personal information into the free text fields in this survey. We will delete any personal information you might insert into the free text fields in order to keep the survey anonymous.

The results of this analysis will be used to steer the development of training materials, and the general communication and dissemination strategy in the Real4Reg project with the goal of facilitating the adoption and implementation of RWD and RWD usage in healthcare decision-making in Europe. The anonymized results of the survey will be published in relevant contexts, e.g., on the Real4Reg website or in a peer-reviewed journal. No further processing of the data for any other purposes outside the scope of this specific context is intended.

Data collection method

For the collection of data in this survey, the external EU survey system "EUSurvey" will be used. For more information on how EUSurvey processes personal data, please see: https://ec.europa.eu/eusurvey/home/privacystatement

This survey will use the "Anonymous survey mode", which means that EUSurvey will not save any personal data such as IP addresses.

Contact information

Publisher and responsible for this survey is the Bundesinstitut für Arzneimittel und Medizinprodukte (BfArM, Federal Institute for Drugs and Medical Devices), more specifically the research group "Pharmakoepidemiologie" of the Research Department.

Contact Information:

Address: Kurt-Georg-Kiesinger-Allee 3, 53175 Bonn, Germany

E-Mail: Real4Reg@bfarm.de

General Information

1 In which country is your primary workplace located?

- Austria
- Belgium
- Bulgaria
- Croatia
- Cyprus
- Czechia
- Denmark
- Estonia
- Finland
- France
- Germany
- Greece
- Hungary
- Ireland
- Italy
- Latvia
- Lithuania
- Luxembourg
- Malta
- Netherlands
- Poland
- Portugal
- Romania
- Slovak Republic
- Slovenia
- Spain
- Sweden
- United Kingdom
- Switzerland
- Other

2 In which other country is your primary workplace located?

3 Which of the following best describes your employer?

If you are active in multiple organizations, please select your primary workplace.

- Regulatory and policy body (e.g. EMA or national competent authorities)
- Health technology assessment body
- Pharmaceutical or medical device company (sponsor)
- Clinical research organization (CRO)
- Consulting company or similar

- Health technology/health data company

4 What best describes your primary domain of work?

Please select the answer which you think best fits your role. Below are descriptions of the categories.

Regulatory & Compliance: Ensuring adherence to standards and regulations
Clinical Development & Research: Design, management, and execution of clinical trials or medical research
Data & Analytics: Handling, analysing, or interpreting data
Technology & Development: Developing new technologies, tools, or methodologies, including AI/ML models or other RWD/RWE related analysis tools
Policy & Strategy: Shaping or advising on policies and strategic directions.
Medical & Scientific Affairs: Medical science liaison activities, medical information, scientific communication, etc.
Consultancy & Advisory: Providing external expertise, often as third-party consultants or advisors in drug development or regulatory areas

- Regulatory & compliance
- Clinical development & research
- Data & analytics
- Technology & development
- Policy & strategy
- Medical & scientific affairs
- Consultancy & advisory

Knowledge about RWD/RWE and AI/ML

5 Please rate your current level of proficiency for each RWD related topic

RWD: real-world data
RWE: real-world evidence
AI: artificial intelligence
ML: machine learning

No knowledge: Completely unfamiliar with the topic
Minimal knowledge: Recognize basic terms or concepts, but not how they apply or function.
Basic knowledge: Understanding of fundamental concepts, can handle simple tasks, but limited hands-on experience and no deeper understanding
Intermediate knowledge: Good understanding with practical experience, confident in common situations.
Advanced knowledge: Deep understanding with significant experience, can navigate complex situations, is sought for advice on the topic by others,
Expert knowledge: Authority in the topic, extensive experience; can teach, mentor and lead in this topic.

	No knowledge	Minimal knowledge	Basic knowledge	Intermediate knowledge	Advanced knowledge	Expert knowledge
**Basic statistical methodology** of clinical trials and observational studies; differences between randomized clinical trials (RCTs) and non-interventional studies.	○	○	○	○	○	○

**RWD sources & management:** Identification and evaluation of RWD sources, access conditions, data sharing regulations; data transformation and quality; synthetic data.	○	○	○	○	○	○
**Technical proficiency:** Familiarity with statistical software and programming relevant to RWD analysis.	○	○	○	○	○	○

**RWD to RWE transformation:** Distilling reliable evidence from RWD, taking into account study design, defining research questions, and statistical approaches; differentiating and combining RCTs and non-interventional trials.	○	○	○	○	○	○
**AI/ML in RWD analysis:** Basics and application of AI /ML tools tailored for RWD; challenges and opportunities.	○	○	○	○	○	○

**Interpretation of findings:** Evaluating and discerning results from RWD-based studies; potential implications.	◯	◯	◯	◯	◯	◯
**Regulatory landscape:** Current regulatory practices and guidelines concerning RWD/RWE utilization.	◯	◯	◯	◯	◯	◯

Opinions regarding RWD/RWE

6 Please indicate how important/useful you think each RWD/RWE related topic will be to you in the future.

RWD: real-world data
RWE: real-world evidence
AI: artificial intelligence
ML: machine learning

Not important at all: Do not see any relevance for the future.
Slightly important: Recognize some relevance or potential, but unsure of its widespread necessity.
Moderately important: Acknowledge its role in the future, it might be handy in some contexts, but not universally.
Important: Predict its consistent need in many situations; it may become a valuable asset.
Very important: Predict it being central in various situations; it will be vital for many and have extensive implications for success.
Absolutely essential: Predict it becoming a paramount skill; indispensable for the majority.

	Not important at all	Slightly important	Moderately important	Important	Very important	Absolutely essential
**Basic statistical methodology and regulatory background** of clinical trials and observational studies; differences between randomized clinical trials (RCTs) and non-interventional studies.	○	○	○	○	○	○

**RWD sources & management:** Identification and evaluation of RWD sources, access conditions, data sharing regulations; data transformation and quality; synthetic data.	○	○	○	○	○	○
**RWD to RWE transformation:** Distilling reliable evidence from RWD, taking into account study design, research questions, and statistical approaches; differentiating and combining RCTs and non-interventional trials.	○	○	○	○	○	○
**AI/ML in RWD analyses**: Basics and application of AI/ML tools tailored for RWD; challenges and opportunities.	○	○	○	○	○	○

**Interpretation of findings:** Evaluating and discerning results from RWD-based studies; potential implications.	○	○	○	○	○	○
**Regulatory landscape:** Current regulatory practices and guidelines concerning RWD /RWE utilization.	○	○	○	○	○	○
**Technical proficiency:** Familiarity with statistical software and programming relevant to RWD analysis.	○	○	○	○	○	○

7 In general, how important do you consider the use of RWD/RWE to support regulatory decision-making and HTA in the future?

RWD: real-world data
RWE: real-world evidence
HTA: health technology assessment

- Not important at all
- Slightly important
- Moderately important
- Important
- Very important
- Absolutely essential

8 In general, how important do you consider the use of AI/ML-based algorithms to support regulatory decision-making and HTA in the future?

AI: artificial intelligence
ML: machine learning
HTA: health technology assessment

- Not important at all
- Slightly important
- Moderately important
- Important
- Very important
- Absolutely essential

Current usage of RWD/RWE and AI/ML

9 Do you already use RWD/RWE for regulatory affairs and/or HTA purposes?

- Yes
- No
- I don't know

10 For which purposes related to regulatory affairs and/or HTA do you already use RWD/RWE?

- Pre-authorization efficacy assessment
- Pre-authorization safety assessment
- Contextualisation: Analysis of disease epidemiology, disease progression, and response to available treatments
- Selection of patients for clinical trials
- Selection of endpoints for clinical trials
- Single-arm trials using historic/external control groups
- Target trial emulation
- Post-authorization efficacy/effectiveness analysis
- Post-authorization safety analysis
- Assessment of unmet medical need
- Indirect treatment comparison
- Other
- I don't know

11 For which other purposes related to regulatory affairs and/or HTA do you already use RWD/RWE?

12 What issues did you encounter when making use of RWD/RWE for regulatory affairs and/or HTA purposes?

- Difficulties accessing data, lack of trusted RWD sources
- Data quality issues (varying data collection standards, doubts whether RWD is fit for purpose)
- Data recording (coding) standardization issues
- Difficulties interpreting studies which use RWD/RWE
- Time and resource constraints to develop and implement new workflows, standard operating procedures (SOPs), etc.
- Shortage of RWD-experienced experts/specialists
- Lack of comprehensive training on RWD/RWE for teams
- Legal and bureaucratic issues (data security and privacy)
- Lack of suitable data analysis tools
- Lack of clear regulatoy guidelines for RWD/RWE usage and submission
- Trust and acceptance issues / cultural resistance
- Other
- I did not encounter any issues
- I don't know

13 What other issues did you encounter when making use of RWD/RWE for regulatory affairs and/or HTA purposes?

14 What are the reasons due to which you are currently not using RWD/RWE for regulatory affairs and/or HTA purposes?

- Difficulties accessing data, lack of trusted RWD sources
- Data quality issues (varying data collection standards, doubts whether RWD is fit for purpose)
- Data recording (coding) standardization issues
- Difficulties interpreting studies which use RWD/RWE
- Time and resource constraints to develop and implement new workflows, standard operating procedures (SOPs), etc.
- Shortage of RWD-experienced experts/specialists
- Lack of comprehensive training on RWD/RWE for teams
- Legal and bureaucratic issues (data security and privacy)
- Lack of suitable data analysis tools
- Lack of clear regulatoy guidelines for RWD/RWE usage and submission
- Trust and acceptance issues/cultural resistance
- Other
- I currently don't require RWD
- I don't know

15 For what other reasons are you currently not using RWD/RWE for regulatory affairs and/or HTA purposes?

16 Do you already make use of AI/ML-based algorithms for the analysis of RWD/RWE related to regulatory affairs and/or HTA?

- Yes
- No
- I don't know

17 For which applications do you already make use of AI/ML-based algorithms for RWD analysis related to regulatory affairs and/or HTA purposes?

- ☐ Subset real-world datasets by user defined criteria
- ☐ Analyse, cluster, and visualize the characteristics of patient subsets
- ☐ Predict drug safety related issues
- ☐ Predict post-marketing effectiveness data
- ☐ Predict drug combination effects
- ☐ Predict drug repurposing potential
- ☐ Construction of synthetic/historical control arms
- ☐ Optimization of clinical trials (choice of endpoints, dosage levels, inclusion/exclusion criteria)
- ☐ Real-time monitoring and data analysis of clinical trials
- ☐ Generation of synthetic (real world) data
- ☐ Data quality control and validation
- ☐ Pharmacovigilance
- ☐ Regulatory compliance and risk management
- ☐ Biomarker discovery and evaluation
- ☐ Other
- ☐ I don't know

18 For which other applications do you already make use of AI/ML-based algorithms for RWD analysis related to regulatory affairs and/or HTA purposes?

19 Which issues did you encounter when making use of AI/ML-based algorithms for RWD analysis for regulatory affairs and/or HTA purposes?

- ☐ No access to AI/ML models at all
- ☐ Model validation issues
- ☐ Model generalization and overfitting issues
- ☐ Lack of (qualitative) training datasets
- ☐ Interpretability issues/explainable AI issues
- ☐ Reproducibility issues
- ☐ Lack of computing power
- ☐ Integration issues (problems integrating the new models into existing IT systems or workflows)
- ☐ Regulatory and compliance challenges (e.g. due to unclear regulatory guidelines or data privacy issues)
- ☐ Acceptance and trust issues towards AI/ML models by other stakeholders
- ☐ Lack of skilled personnel
- ☐ Other
- ☐ I did not encounter any issues
- ☐ I don't know

20 What other issues did you encounter when making use of AI/ML based algorithms for RWD analysis for regulatory affairs and/or HTA purposes?

21 What are the reasons due to which you are currently not employing AI/ML-based algorithms for RWD analysis for regulatory affairs and/or HTA purposes?

- ☐ No access to AI/ML models at all
- ☐ Model validation issues
- ☐ Model generalization and overfitting issues
- ☐ Lack of (relevant) training datasets
- ☐ Interpretability issues/explainable AI issues
- ☐ Reproducibility issues
- ☐ Integration issues (problems integrating the new models into existing IT systems or workflows)
- ☐ Regulatory and compliance challenges (e.g. due to unclear regulatory guidelines or data privacy issues)
- ☐ Acceptance and trust issues towards AI/ML models by other stakeholders
- ☐ Lack of skilled personnel
- ☐ Other
- ☐ I currently don't require AI/ML-based algorithms

- [ ] Lack of computing power
- [ ] I don't know

22 For which other reasons are you currently not employing AI/ML based algorithms for RWD analysis for regulatory affairs and/or HTA purposes?

23 Do you have access to external RWD sets?

- ( ) Yes
- ( ) No
- ( ) I don't know

24 What types of RWD sets do you have access to?

- [ ] Electronic health records (EHRs)
- [ ] Disease or patient registries
- [ ] Medical claims and billing data
- [ ] Prescription records (pharmacy dispensing data)
- [ ] Patient-generated data (e.g. from wearables or apps)
- [ ] Social media based data
- [ ] Patient-powered research networks
- [ ] Laboratory results (e.g. imaging data)
- [ ] Biobanks or genetic databases
- [ ] Other
- [ ] I don't know

25 Which other types of external RWD sets do you have access to?

26 Which RWD sources would you like to use, but currently cannot?

- [ ] Electronic health records (EHRs)
- [ ] Disease or patient registries
- [ ] Medical claims and billing data
- [ ] Prescription records (pharmacy dispensing data)
- [ ] Patient-generated data (e.g. from wearables or apps)
- [ ] Social media based data
- [ ] Patient-powered research networks
- [ ] Laboratory results (e.g. imaging data)
- [ ] Biobanks or genetic databases
- [ ] Other
- [ ] I don't know

27 Which other types of RWD sources would you like to use, but currently cannot?

28 Are you already consulting (draft) guidelines, or data frameworks, or similar types of guidance documents about the use of RWD/RWE for regulatory affairs and/or HTA purposes?

- Yes
- No
- I don't know

29 In your opinion, in which aspects do the currently available guidelines have gaps?

- Data standards: Quality criteria, reliability, relevance, recording
- Study design methods
- Data analysis methods
- Use of AI/ML-based algorithms for data analysis
- Integration of RWD from multiple sources
- Data security and privacy issues
- Synthetic data
- Study submission and reporting during the regulatory process
- Study submission and reporting during the HTA process
- Global harmonization
- EU-wide harmonization
- Best practice examples
- Other
- I don't know

30 What other gaps exist in the current guidelines?

31 Do you already make use of a common data model (CDM) when working with RWD?

- Yes
- No
- I don't know

32 Which CDM do you use when working with RWD?

- PCORnet
- FDA Sentinel
- i2b2
- OMOP
- Sentinel CIDA
- Aetion
- EU-ADR
- IMI-PROTECT
- CNODES
- Other
- I don't know

33 Which other CDM do you use when working with RWD?

Interest in RWD/RWE and AI/ML

34 Are you interested in (AI/ML-based) tools for the analysis of RWD/RWE?

- Yes
- No

35 In (AI/ML-based) tools for which applications are you particularly interested in?

- Subset real-world datasets by user defined criteria

- ☐ Analyse, cluster, and visualize the characteristics of patient subsets
- ☐ Predict drug safety related issues
- ☐ Predict post-marketing effectiveness data
- ☐ Predict drug combination effects
- ☐ Predict drug repurposing potential
- ☐ Construction of synthetic/historical control arms
- ☐ Optimization of clinical trials (choice of endpoints, dosage levels, inclusion/exclusion criteria)
- ☐ Real-time monitoring and data analysis of clinical trials
- ☐ Generation of synthetic (real-world)-data
- ☐ Data quality control and validation
- ☐ Pharmacovigilance
- ☐ Regulatory compliance and risk management
- ☐ Biomarker discovery and evaluation
- ☐ Other
- ☐ I don't know

36 In (AI/ML-based) tools for which other applications/use cases are you particularly interested in?

37 Are you interested in participating in training sessions or educational programs to learn more about the use RWD /RWE for regulatory affairs and/or HTA?

- ○ Yes
- ○ No

38 In what format would you prefer the educational programs to take place?

Use drag&drop or the up/down buttons to change the order or accept the initial order.

- Video tutorials
- Blended learning: Combination of in-person events, online events, and self-learning
- (Self-paced) online courses
- Online training (seminars, workshops)
- In-person training (seminars, workshops)

39 Are you interested in good practice examples related to the use of RWD/RWE for regulatory affairs and/or HTA purposes?

- ○ Yes
- ○ No

40 You are interested in good practice examples using RWD/RWE. For which purposes specifically?

- ☐ Pre-authorization efficacy assessment
- ☐ Pre-authorization safety assessment
- ☐ Contextualization: Analysis of disease epidemiology, disease progression, and response to available treatments
- ☐ Selection of patients for clinical trials
- ☐ Selection of endpoints for clinical trials
- ☐ Single-arm trials using historic/external control groups
- ☐
- ☐ Post-authorization efficacy/effectiveness analysis
- ☐ Post-authorization safety analysis
- ☐ Assessment of unmet medical need
- ☐ Indirect treatment comparison
- ☐ Other
- ☐ I don't know

Target trial emulation

41 For which *other* purposes are you interested in good practice examples using RWD/RWE?

End

42 We value your insights and experiences in the realm of RWD and RWE implementation. In this open-ended question, we invite you to share your perspectives on two key aspects:

- Obstacles and challenges: What, in your opinion, are the most significant challenges or obstacles that currently hinder the effective implementation of RWD and RWE in drug regulatory decision-making and health technology assessment (HTA)? Please feel free to elaborate on any technical, organizational, regulatory, or other types of barriers you have encountered or anticipate.
- Additional remarks: Is there anything else you would like to share with us regarding the use of RWD and RWE? This could include suggestions for improvement, areas needing more attention, or any other comments you believe are pertinent to our project's objectives.

Your input is invaluable in guiding the development of standards, methods, and training concepts for the use of RWD and RWE. Thank you for taking the time to contribute your expertise and insights.

Thank you

Thank you very much for completing this survey. Your input is greatly appreciated.

Please click the **submit** button to submit your answers.

You can stay up-to-date on Real4Reg by following the links on the sidebar.

Real4Reg Survey - Academia

Introduction

Welcome to the Real4Reg Survey on Real-World Data and Real-World Evidence use in Regulatory Affairs and Health Technology Assessment

Background

As defined by the European Medicines Agency (EMA), real-world data (RWD) encompasses “routinely collected data relating to a patient's health status or the delivery of health care from a variety of sources other than traditional clinical trials.” Real-world evidence (RWE), on the other hand, is “information derived from analysis of RWD.” While traditionally mainly used for pharmacovigilance purposes, RWE can also be used for pre-authorisation efficacy and safety studies, to supplement and improve the efficacy of clinical trials, and to conduct trials, which would otherwise be very difficult to run. In other words, the use of RWD can increase the safety and efficacy of drugs available to patients, while at the same time improving the drug development process by making it more cost-effective and faster. So far though, RWD and RWE currently only play a limited role in regulatory decision-making and health technology assessment (HTA). The Real4Reg Project aims to establish their values in these areas.

The Real4Reg Project

Real4Reg is a consortium consisting of ten partners from six different European countries with experience in the field of RWD analyses, including experts from regulatory agencies and HTA bodies (BfArM, DKMA, Infarmed), academia (Fraunhofer, UEF, CSC, AU, DZNE), and patient organisations (EUpALS, EIWH).

To support and facilitate the widespread adoption of RWD/RWE usage in regulatory affairs and HTA, we are developing standards for RWD/RWE use, including artificial intelligence (AI) and machine learning (ML) tools to manage, analyse, and interpret this data. These tools will be validated through a series of good practice examples on currently highly relevant research topics in the field of drug safety, efficacy, and clinical research. We are also creating training modules to fill existing gaps and enhance the RWD/RWE skills within the regulatory and industry sectors. For an in-depth look at our work, visit our website at https://www.real4reg.eu/.

Aim of this survey

This survey is designed to gather a well-rounded view of stakeholders' requirements, knowledge, capacities, and opinions on RWD/RWE. We aim to identify knowledge gaps that could hinder the successful implementation of RWD/RWE in regulatory and HTA processes. Academic research is at the forefront of innovation and the availability of RWD has the potential to advance research in various areas, which is why we are very interested in your opinions and experiences on this matter.

How to complete the survey
Please only complete this survey if you are currently working for an academic institution in a research area related to pharmaceutical- and medical device research for an academic institution.

Answering the survey will take approximately 10-12 minutes. When answering questions in this survey, please make sure to click the small question mark (?) symbol next to some questions.

By participating in this survey, you are playing a crucial role in our journey. Your expertise and perspective are invaluable assistance to our work.
Thank you for participating!

Data protection

Data collection within this survey
Your data is collected for the purpose of scientifically analysing the current state of real-world data (RWD) and real-world evidence (RWE) usage, expertise, and the stakeholder's general opinions towards this topic.

All data collected as part of this survey is anonymized. This means, your responses will not be identifiable to you as an individual. All data gathered during this survey will be aggregated and analysed. In order to stay anonymous, please do not insert any personal information into the free text fields in this survey. We will delete any personal information you might insert into the free text fields in order to keep the survey anonymous.

The results of this analysis will be used to steer the development of training materials, and the general communication and dissemination strategy in the Real4Reg project with the goal of facilitating the adoption and implementation of RWD and RWD usage in healthcare decision-making in Europe. The anonymized results of the survey will be published in relevant contexts, e.g., on the Real4Reg website or in a peer-reviewed journal. No further processing of the data for any other purposes outside the scope of this specific context is intended.

Data collection method
For the collection of data in this survey, the external EU survey system "EUSurvey" will be used. For more information on how EUSurvey processes personal data, please see: https://ec.europa.eu/eusurvey/home /privacystatement This survey will use the "Anonymous survey mode", which means that EUSurvey will not save any personal data such as IP addresses.

Contact information
Publisher and responsible for this survey is the Bundesinstitut für Arzneimittel und Medizinprodukte (BfArM, Federal Institute for Drugs and Medical Devices), more specifically the research group "Pharmakoepidemiologie" of the Research Department.

Contact Information:
Address: Kurt-Georg-Kiesinger-Allee 3, 53175 Bonn, Germany
E-Mail: Real4Reg@bfarm.de

General Information

1 In which country is your primary workplace located?

- Austria
- Belgium
- Bulgaria
- Croatia
- Cyprus
- Czechia
- Denmark
- Estonia
- Finland
- France
- Germany
- Greece
- Hungary
- Ireland
- Italy
- Latvia
- Lithuania
- Luxembourg
- Malta
- Netherlands
- Poland
- Portugal
- Romania
- Slovak Republic
- Slovenia
- Spain
- Sweden
- United Kingdom
- Switzerland
- Other

2 In which other country is your primary workplace located?

3 What best describes the institution you currently work for?

If you are active in multiple organizations, please select your primary workplace.

- Public university
- Private university
- Research organization or research institute
- Government research facility (or research department of a government body)
- Hospital with academic affiliation
- Independent research consultant

- Other educational institution
- Other health care facility

4 What best describes your area of expertise in which you primarily work in?

Please select the answer which you think best fits your role.

- (Health) informatics
- Biostatistics and data science
- Epidemiology and public health
- Health economics
- Pharmacology and toxicology
- Biomedical research and biotechnology
- Genomics or other –omics
- Medical ethics and legal/regulatory aspects of health research
- Clinical research including clinical trials
- Other

5 What is your highest academic degree?

- High school degree
- Bachelor’s degree
- Master’s degree
- PhD
- (Tenured) Professorship

Knowledge about RWD/RWE and AI/ML

6 Please rate your current level of proficiency for each RWD related topic

RWD: real-world data
RWE: real-world evidence
AI: artificial intelligence
ML: machine learning

No knowledge: Completely unfamiliar with the topic
Minimal knowledge: Recognize basic terms or concepts, but not how they apply or function.
Basic knowledge: Understanding of fundamental concepts, can handle simple tasks, but limited hands-on experience and no deeper understanding
Intermediate knowledge: Good understanding with practical experience, confident in common situations.
Advanced knowledge: Deep understanding with significant experience, can navigate complex situations, is sought for advice on the topic by others,
Expert knowledge: Authority in the topic, extensive experience; can teach, mentor and lead in this topic.

	No knowledge	Minimal knowledge	Basic knowledge	Intermediate knowledge	Advanced knowledge	Expert knowledge
**Basic statistical methodology** of clinial trials and observational studies; differences between randomized clinical trials (RCTs) and non-interventional studies.	○	○	○	○	○	○

**RWD sources & management:** Identification and evaluation of RWD sources, access conditions, data sharing regulations; data transformation and quality; synthetic data.	○	○	○	○	○	○
**Technical proficiency:** Familiarity with statistical software and programming relevant to RWD analysis.	○	○	○	○	○	○

**RWD to RWE transformation:** Distilling reliable evidence from RWD, taking into account study design, research questions, and statistical approaches; differentiating and combining RCTs and non-interventional trials.	○	○	○	○	○	○
**AI/ML in RWD analysis:** Basics and application of AI /ML tools tailored for RWD; challenges and opportunities.	○	○	○	○	○	○

**Interpretation of findings:** Evaluating and discerning results from RWD-based studies; potential implications.						
**Regulatory landscape:** Current regulatory practices and guidelines concerning RWD/RWE utilization.						

7 Please indicate how important/useful you think each RWD/RWE related topic will be to you in the future.

RWD: real-world data
RWE: real-world evidence
AI: artificial intelligence
ML: machine learning

Not important at all: Do not see any relevance for the future.
Slightly important: Recognize some relevance or potential, but unsure of its widespread necessity.
Moderately important: Acknowledge its role in the future; it might be handy in some contexts, but not universally.
Important: Predict its consistent need in many situations; it may become a valuable asset.
Very important: Predict it being central in various situations; it will be vital for many and have extensive implications for success.
Absolutely essential: Predict it becoming a paramount skill; indispensable for the majority.

	Not important at all	Slightly important	Moderately important	Important	Very important	Absolutely essential
**Basic statistical methodology and regulatory background** of clinical trials and observational studies; differences between randomized clinical trials (RCTs) and non-interventional studies.	○	○	○	○	○	○

**RWD sources & management:** Identification and evaluation of RWD sources, access conditions, data sharing regulations; data transformation and quality; synthetic data.	◯	◯	◯	◯	◯	◯
**RWD to RWE transformation:** Distilling reliable evidence from RWD, taking into account study design, research questions, and statistical approaches; differentiating and combining RCTs and non-interventional trials.	◯	◯	◯	◯	◯	◯
**AI/ML in RWD analyses**: Basics and application of AI/ML tools tailored for RWD; challenges and opportunities.	◯	◯	◯	◯	◯	◯

**Interpretation of findings:** Evaluating and discerning results from RWD-based studies; potential implications.	○	○	○	○	○	○
**Regulatory landscape:** Current regulatory practices and guidelines concerning RWD /RWE utilization.	○	○	○	○	○	○
**Technical proficiency:** Familiarity with statistical software and programming relevant to RWD analysis.	○	○	○	○	○	○

8 In general, how important do you consider the use of RWD/RWE to support regulatory decision-making and HTA in the future?

RWD: real-world data
RWE: real-world evidence
HTA: health technology assessment

- Not important at all
- Slightly important
- Moderately important
- Important
- Very important
- Absolutely essential

9 In general, how important do you consider the use of AI/ML-based algorithms to support regulatory decision-making and HTA in the future?

AI: artificial intelligence
ML: machine learning
HTA: health technology assessment

- Not important at all
- Slightly important
- Moderately important
- Important
- Very important
- Absolutely essential

Current usage of RWD/RWE and AI/ML

10 Do you use RWD/RWE in your research?

- Yes
- No
- I don't know

11 For what purposes are you using RWD/RWE?

- [ ] Exploratory research and hypothesis generation
- [ ] Epidemiological studies
- [ ] Pharmacoeconomic research
- [ ] Development of data analysis methodologies and tools
- [ ] Educational purposes
- [ ] Other

12 For which other purposes are you using RWD/RWE?

13 What issues did you encounter when using RWD/RWE?

- [ ] Difficulties accessing data, lack of trusted RWD sources
- [ ] Legal and bureaucratic issues (data security and privacy)

- [ ] Data quality issues (varying data collection standards, doubts whether RWD is fit for purpose)
- [ ] Lack of suitable data analysis tools
- [ ] Data recording (coding) standardization issues
- [ ] Lack of clear regulatory guidelines for RWD/RWE usage and submission
- [ ] Difficulties interpreting studies which use RWD/RWE
- [ ] Trust and acceptance issues/cultural resistance
- [ ] Time and resource constraints to develop and implement new workflows, standard operating procedures (SOPs), etc.
- [ ] Other
- [ ] Shortage of RWD-experienced experts/specialists
- [ ] I did not encounter any issues
- [ ] Lack of comprehensive training on RWD/RWE for teams
- [ ] I don't know

14 What other issues did you encounter when making use of RWD/RWE?

15 What are the reasons due to which you are currently not using RWD/RWE?

- [ ] Difficulties accessing data, lack of trusted RWD sources
- [ ] Legal and bureaucratic issues (data security and privacy)
- [ ] Data quality issues (varying data collection standards, doubts whether RWD is fit for purpose)
- [ ] Lack of suitable data analysis tools
- [ ] Data recording (coding) standardization issues
- [ ] Lack of clear regulatory guidelines for RWD/RWE usage and submission
- [ ] Difficulties interpreting studies which use RWD/RWE
- [ ] Trust and acceptance issues/cultural resistance
- [ ] Time and resource constraints to develop and implement new workflows, standard operating procedures (SOPs), etc.
- [ ] Other
- [ ] Shortage of RWD-experienced experts/specialists
- [ ] I currently don't require RWD
- [ ] Lack of comprehensive training on RWD/RWE for teams
- [ ] I don't know

16 For what other reasons are you currently not using RWD/RWE?

17 Do you already make use of AI/ML based algorithms for the analysis of RWD/RWE?

- ( ) Yes
- ( ) No
- ( ) I don't know

18 For which applications/purposes/use cases do you already make use of AI/ML-based algorithms for RWD analysis?

19 Which issues did you encounter when making use of AI/ML-based algorithms for RWD analysis?

- [ ] No access to AI/ML models at all
- [ ] Integration issues (problems integrating the new models into existing IT systems or workflows)

- [ ] Model validation issues
- [ ] Model generalization and overfitting issues
- [ ] Lack of (relevant) training datasets
- [ ] Interpretability issues/explainable AI issues
- [ ] Reproducibility issues
- [ ] Lack of computing power
- [ ] Regulatory and compliance challenges (e.g. due to unclear regulatory guidelines or data privacy issues)
- [ ] Acceptance and trust issues towards AI/ML models by other stakeholders
- [ ] Lack of skilled personnel
- [ ] Other
- [ ] I did not encounter any issues
- [ ] I don't know

20 What other issues did you encounter when making use of AI/ML based algorithms for RWD analysis for regulatory affairs and/or HTA purposes?

21 What are the reasons due to which you are currently not employing AI/ML based algorithms for RWD analysis?

- [ ] No access to AI/ML models at all
- [ ] Model validation issues
- [ ] Model generalization and overfitting issues
- [ ] Lack of (relevant) training datasets
- [ ] Interpretability issues/explainable AI issues
- [ ] Reproducibility issues
- [ ] Lack of computing power
- [ ] Integration issues (problems integrating the new models into existing IT systems or workflows)
- [ ] Regulatory and compliance challenges (e.g. due to unclear regulatory guidelines or data privacy issues)
- [ ] Acceptance and trust issues towards AI/ML models by other stakeholders
- [ ] Lack of skilled personnel
- [ ] Other
- [ ] I currently don't require AI/ML-based algorithms
- [ ] I don't know

22 For which other reasons are you currently not employing AI/ML based algorithms for RWD analysis?

23 Do you have access to external RWD sets?

- ○ Yes
- ○ No
- ○ I don't know

24 What types of RWD sets do you have access to?

- [ ] Electronic health records (EHRs)
- [ ] Disease or patient registries
- [ ] Medical claims and billing data
- [ ] Prescription records (pharmacy dispensing data)
- [ ] Patient-generated data (e.g. from wearables or apps)
- [ ] Social media based data
- [ ] Patient-powered research networks
- [ ] Laboratory results (e.g. imaging data)
- [ ] Biobanks or genetic databases

- [ ] Other
- [ ] I don't know

25 Which other types of external RWD sets do you have access to?

26 Which RWD sources would you like to use, but currently cannot?

- [ ] Electronic health records (EHRs)
- [ ] Disease or patient registries
- [ ] Medical claims and billing data
- [ ] Prescription records (pharmacy dispensing data)
- [ ] Patient-generated data (e.g. from wearables or apps)
- [ ] Social media based data
- [ ] Patient-powered research networks
- [ ] Laboratory results (e.g. imaging data)
- [ ] Biobanks or genetic databases
- [ ] Other
- [ ] I don't know

27 Which other types of RWD sources would you like to use, but currently cannot?

28 Do you already make use of a common data model (CDM) when working with RWD?

- ( ) Yes
- ( ) No
- ( ) I don't know

29 Which CDM do you use when working with RWD?

- [ ] PCORnet
- [ ] FDA Sentinel
- [ ] i2b2
- [ ] OMOP
- [ ] Sentinel CIDA
- [ ] Aetion
- [ ] EU-ADR
- [ ] IMI-PROTECT
- [ ] CNODES
- [ ] Other
- [ ] I don't know

30 Which other CDM do you use when working with RWD?

Interest in RWD/RWE and AI/ML

31 Are you interested in (AI/ML-based) tools for the analysis of RWD/RWE?

- ( ) Yes
- ( ) No

32 In (AI/ML-based) tools for which applications/use cases are you particularly interested in?

33 Are you interested in participating in training sessions or educational programs to learn more about the use RWD /RWE?

- Yes
- No

34 In what format would you prefer the educational programs to take place?

Use drag&drop or the up/down buttons to change the order or accept the initial order.

- (Self-paced) online courses
- Online training (seminars, workshops)
- In-person training (seminars, workshops)
- Video tutorials
- Blended learning: Combination of in-person eents, online events, and self-training

35 Are you interested in good practice examples related to the use of RWD/RWE for regulatory affairs and/or HTA purposes?

- Yes
- No

36 You are interested in good practice examples using RWD/RWE. For which purposes specifically?

- Pre-authorization efficacy assessment
- Pre-authorization safety assessment
- Contextualisation: Analysis of disease epidemiology, disease progression, and response to available treatments
- Selection of patients for clinical trials
- Selection of endpoints for clinical trials
- Single-arm trials using historic/external control groups
- Target trial emulation
- Post-authorization efficacy/effectiveness analysis
- Post-authorization safety analysis
- Assessment of unmet medical need
- Indirect treatment comparison
- Other
- I don't know

37 For which *other* purposes are you specifically interested in good practice examples using RWD/RWE?

End

38 We value your insights and experiences in the realm of RWD and RWE implementation. In this open-ended question, we invite you to share your perspectives on two key aspects:

- Obstacles and challenges: What, in your opinion, are the most significant challenges or obstacles that currently hinder the effective implementation of RWD and RWE in drug regulatory decision-making and health technology assessment (HTA)? Please feel free to elaborate on any technical, organizational, regulatory, or other types of barriers you have encountered or anticipate.
- Additional remarks: Is there anything else you would like to share with us regarding the use of RWD and RWE? This could include suggestions for improvement, areas needing more attention, or any other comments you believe are pertinent to our project's objectives.

Your input is invaluable in guiding the development of standards, methods, and training concepts for the use of RWD and RWE. Thank you for taking the time to contribute your expertise and insights.

Thank you

Thank you very much for completing this survey. Your input is greatly appreciated.

Please click the **submit** button to submit your answers.

You can stay up-to-date on Real4Reg by following the links on the sidebar.

Real4Real Survey - Payers

Introduction

Welcome to the Real4Reg Survey on Real-World Data and Real-World Evidence use in Regulatory Affairs and Health Technology Assessment

Background

As defined by the European Medicines Agency (EMA), real-world data (RWD) encompasses “routinely collected data relating to a patient's health status or the delivery of health care from a variety of sources other than traditional clinical trials.” Real-world evidence (RWE), on the other hand, is “information derived from analysis of RWD.” While traditionally mainly used for pharmacovigilance purposes, RWE can also be used for pre-authorisation efficacy and safety studies to supplement and improve the efficacy of clinical trials and to conduct trials, which would otherwise be very difficult to run. In other words, the use of RWD can increase the safety and efficacy of drugs available to patients, while at the same time improve the drug development process by making it more cost-effective and faster. So far though, RWD and RWE currently only play a limited role in regulatory decision-making and Health Technology Assessment (HTA). The Real4Reg Project aims to establish their values in these areas.

The Real4Reg Project

Real4Reg is a consortium consisting of ten partners from six different European countries with experience in the field of RWD analyses, including experts from regulatory agencies and HTA bodies (BfArM, DKMA, Infarmed), academia (Fraunhofer, UEF, CSC, AU, DZNE), and patient organisations (EUpALS, EIWH).

To support and facilitate the widespread adoption of RWD/RWE usage in regulatory affairs and HTA, we are developing standards for RWD/RWE use, including AI/ML tools to manage, analyse, and interpret this data. These tools will be validated through a series of good practice examples on currently highly relevant research topics in the field of drug safety, efficacy, and clinical research. We are also creating training modules to fill existing gaps and enhance the RWD/RWE skills within the regulatory and industry sectors. For an in-depth look at our work, visit our website at https://www.real4reg.eu/.

Aim of this survey

This survey is designed to gather a well-rounded view of stakeholders' requirements, knowledge, capacities, and opinions on RWD/RWE. We aim to identify knowledge gaps that could hinder the successful implementation of RWD/RWE in regulatory and HTA processes.

Payer organizations play a central role within the healthcare system and interact with nearly all other stakeholders regularly. Especially in regards to the RWD ecosystem, payer organizations play a key role. In other words, payers have a good overview of current trends and issues, which is why we are very interested in your views and experiences and hope to engage with you further along the way.

How to complete the survey

Please only complete this survey if you are currently working for a healthcare insurance company or another payer organization within the healthcare system.

Answering the survey will take approximately 10-12 minutes. When answering questions in this survey, please make sure to click the small question mark (?) symbol next to some questions.

By participating in this survey, you are playing a crucial role in our journey. Your expertise and perspective are invaluable assistance to our work.
Thank you for participating!

Data protection

Data collection within this survey

Your data is collected for the purpose of scientifically analysing the current state of Real-world data (RWD) and real-world evidence (RWE) usage, expertise, and the stakeholder's general opinions towards this topic.

All data collected as part of this survey is anonymized. This means, your responses will not be identifiable to you as an individual. All data gathered during this survey will be aggregated and analyzed. In order to stay anonymous, please do not insert any personal information into the free text fields in this survey. We will delete any personal information you might insert into the free text fields in order to keep the survey anonymous.

The results of this analysis will be used to steer the development of training materials, and the general communication and dissemination strategy in the Real4Reg project with the goal of facilitating the adoption and implementation of RWD and RWD usage in healthcare decision-making in Europe. The anonymized results of the survey will be published in relevant contexts, e.g., on the Real4Reg website or in a peer-reviewed journal. No further processing of the data for any other purposes outside the scope of this specific context is intended.

Data collection method

For the collection of data in this survey, the external EU survey system “EUSurvey” will be used. For more information on how EUSurvey processes personal data, please see: https://ec.europa.eu/eusurvey/home/privacystatement
This survey will use the “Anonymous survey mode”, which means that EUSurvey will not save any personal data such as IP addresses.

Contact information

Publisher and responsible for this survey is the Bundesinstitut für Arzneimittel und Medizinprodukte (BfArM, Federal Institute for Drugs and Medical Devices), more specifically the research group

"Pharmakoepidemiologie" of the Research Department.

Contact Information:
Address: Kurt-Georg-Kiesinger-Allee 3, 53175 Bonn, Germany
E-Mail: Real4Reg@bfarm.de

General Information

1 In which country is your primary workplace located?

- ○ Austria
- ○ Belgium
- ○ Bulgaria
- ○ Croatia
- ○ Cyprus
- ○ Czechia
- ○ Denmark
- ○ Estonia
- ○ Finland
- ○ France
- ○ Germany
- ○ Greece
- ○ Hungary
- ○ Ireland
- ○ Italy
- ○ Latvia
- ○ Lithuania
- ○ Luxembourg
- ○ Malta
- ○ Netherlands
- ○ Poland
- ○ Portugal
- ○ Romania
- ○ Slovak Republic
- ○ Slovenia
- ○ Spain
- ○ Sweden
- ○ United Kingdom
- ○ Switzerland
- ○ Other

2 In which other country is your primary workplace located?

[ ]

3 What is the size of the organization you work for (number of insured members)?

- ○ ≤10,000

- 10,001-100,000
- 100,001-500,000
- 500,001-1,000,000
- 1,000,001-10,000,00
- ≥10,000,001
- I don't know

4 Is your organization public or private?

- Public
- Private
- Mixed
- I don't know

5 Which department or functional area within your organization does your role best align with?

Please select the answer which you think best fits your role.

- Policy & Planning
- Claims & Coverage
- Health Technology Assessment (HTA)
- Data Analysis & Informatics
- Finance & Risk Management
- Regulatory Compliance & Legal
- Marketing & Communications
- Human Resources & Administration
- Customer Service & Support
- Other

Knowledge about RWD/RWE and AI/ML

6 Please rate your current level of proficiency for each RWD related topic

RWD: real-world data
RWE: real-world evidence
AI: artificial intelligence
ML: machine learning

No knowledge: Completely unfamiliar with the topic.
Minimal knowledge: Recognize basic terms or concepts, but not how they apply or function.
Basic knowledge: Understanding of fundamental concepts, can handle simple tasks, but limited hands-on experience and no deeper understanding.
Intermediate knowledge: Good understanding with practical experience, confident in common situations.
Advanced knowledge: Deep understanding with significant experience, can navigate complex situations, is sought for advice on the topic by others.
Expert knowledge: Authority in the topic, extensive experience; can teach, mentor and lead in this topic.

	No knowledge	Minimal knowledge	Basic knowledge	Intermediate knowledge	Advanced knowledge	Expert knowledge
**Basic statistical methodology** of clinial trials and observational studies; differences between randomized clinical trials (RCTs) and non-interventional studies.	○	○	○	○	○	○
**RWD sources & management:**						

Identification and evaluation of RWD sources, access conditions, data sharing regulations, data transformation and quality, synthetic data.	○	○	○	○	○	○
**Technical proficiency:** Familiarity with statistical software and programming relevant to RWD analysis.	○	○	○	○	○	○
**RWD to RWE transformation:** Distilling reliable evidence from RWD, taking into account study design, defining research questions, and statistical approaches; differentiating	○	○	○	○	○	○

and combining RCTs and non-interventional trials.						
**AI/ML in RWD analysis:** Basics and and application of AI /ML tools tailored for RWD; challenges and opportunities.						
**Interpretation of findings:** Evaluating and discerning results from RWD-based studies; potential implications.						
**Regulatory landscape:** Current regulatory practices and guidelines concerning RWD/RWE utilization.						

7 Please indicate how important/useful you think each RWD/RWE related topic will be to you in the future.

RWD: real-world data
RWE: real-world evidence
RCT: randomized clinical trial
AI: artificial intelligence
ML: machine learning

Not important at all: Do not see any relevance for the future.
Slightly important: Recognize some relevance or potential, but unsure of its widespread necessity.
Moderately important: Acknowledge its role in the future, it might be handy in some contexts, but not universally.
Important: Predict its consistent need in many situations; it may become a valuable asset.
Very important: Predict it being central in various situations; it will be vital for many and have extensive implications for success.
Absolutely essential: Predict it becoming a paramount skill; indispensable for the majority.

	Not important at all	Slightly important	Moderately important	Important	Very important	Absolutely essential
**Basic statistical methodology and regulatory background** of clinial trials and observational studies.	○	○	○	○	○	○
**RWD sources & management:** Identification and evaluation of RWD sources, access conditions, data sharing regulations, data transformation and quality, synthetic data.	○	○	○	○	○	○

**RWD to RWE transformation:** Distilling reliable evidence from RWD, taking into account study design, research questions, and statistical approaches; differentiating and combining RCTs and non-interventional trials.	○	○	○	○	○	○
**AI/ML in RWD analyses**: Basics and application of AI/ML tools tailored for RWD; challenges and opportunities	○	○	○	○	○	○
**Interpretation of findings:** Evaluating and discerning results from RWD-based studies; potential implications.	○	○	○	○	○	○
**Regulatory landscape:** Current regulatory practices and guidelines	○	○	○	○	○	○

concerning RWD /RWE utilization.						
**Technical proficiency:** Familiarity with statistical software and programming relevant to RWD analysis.	◯	◯	◯	◯	◯	◯

8 In general, how important do you consider the use of RWD/RWE to support decision-making in the future?

- Not important at all
- Slightly important
- Moderately important
- Important
- Very important
- Absolutely essential

9 In general, how important do you consider the use of AI/ML-based algorithms to support decision-making in the future?

- Not important at all
- Slightly important
- Moderately important
- Important
- Very important
- Absolutely essential

Current usage of RWD/RWE and AI/ML

10 Does your organization have a research department?

- Yes
- No
- I don't know

11 Is your organization actively involved in the HTA process?

HTA: Health Technology Assessment

- Yes
- No
- I don't know

12 Do you already make use of RWD/RWE?

- Yes
- No
- I don't know

13 For which purposes do you already use RWD/RWE?

14 What issues did you encounter when making use of RWD/RWE?

- Difficulties accessing data, lack of trusted RWD sources
- Legal and bureaucratic issues (data security and privacy)
- Data quality issues (varying data collection standards, doubts whether RWD is fit for purpose)
- Lack of suitable data analysis tools
- Data recording (coding) standardization issues
- Lack of clear regulatory guidelines for RWD/RWE usage and submission

- [ ] Difficulties interpreting studies which use RWD/RWE
- [ ] Trust and acceptance issues/cultural resistance
- [ ] Time and resource constraints to develop and implement new workflows, standard operating procedures (SOPs), etc.
- [ ] Other
- [ ] Shortage of RWD-experienced experts/specialists
- [ ] I did not enounter any issues
- [ ] Lack of comprehensive training on RWD/RWE for teams
- [ ] I don't know

15 What other issues did you encounter when making use of RWD/RWE?

16 What are the reasons due to which you are currently not using RWD/RWE?

- [ ] Difficulties accessing data, lack of trusted RWD sources
- [ ] Legal and bureaucratic issues (data security and privacy)
- [ ] Data quality issues (varying data collection standards, doubts whether RWD is fit for purpose)
- [ ] Lack of suitable data analysis tools
- [ ] Data recording (coding) standardization issues
- [ ] Lack of clear regulatory guidelines for RWD/RWE usage and submission
- [ ] Difficulties interpreting studies which use RWD/RWE
- [ ] Trust and acceptance issues/cultural resistance
- [ ] Time and resource constraints to develop and implement new workflows, SOPs, etc.
- [ ] Other
- [ ] Shortage of RWD-experienced experts/specialists
- [ ] I currently don't require RWD
- [ ] Lack of comprehensive training on RWD/RWE for teams
- [ ] I don't know

17 For what other reasons are you currently not using RWD/RWE?

18 Do you already make use of AI/ML based algorithms for the analysis of RWD/RWE?

- ( ) Yes
- ( ) No
- ( ) I don't know

19 For which applications/use cases do you already make use of AI/ML-based algorithms for data analysis?

20 Which issues did you encounter when making use of AI/ML-based algorithms for RWD analysis?

- [ ] No access to AI/ML models at all
- [ ] Integration issues (problems integrating the new models into existing IT systems or workflows)
- [ ] Model validation issues
- [ ] Regulatory and compliance challenges (e.g. due to unclear regulatory guidelines or data privacy issues)
- [ ] Model generalization and overfitting issues
- [ ] Acceptance and trust issues towards AI/ML models by other stakeholders
- [ ] Lack of (relevant) training datasets
- [ ] Lack of skilled personnel
- [ ]
- [ ] Other

- Interpretability issues/explainable AI issues
- [ ] Reproducibility issues
- [ ] Lack of computing power
- [ ] I did not encounter any issues
- [ ] I don't know

21 What other issues did you encounter when making use of AI/ML based algorithms for RWD analysis?

22 What are the reasons due to which you are currently not employing AI/ML based algorithms for RWD analysis?

- [ ] No access to AI/ML models at all
- [ ] Model validation issues
- [ ] Model generalization and overfitting issues
- [ ] Lack of (relevant) training datasets
- [ ] Interpretability issues/explainable AI issues
- [ ] Reproducibility issues
- [ ] Lack of computing power
- [ ] Integration issues (problems integrating the new models into existing IT systems or workflows)
- [ ] Regulatory and compliance challenges (e.g. due to unclear regulatory guidelines or data privacy issues)
- [ ] Acceptance and trust issues towards AI/ML models by other stakeholders
- [ ] Lack of skilled personnel
- [ ] Other
- [ ] I currently don't require AI/ML-based algorithms
- [ ] I don't know

23 For which other reasons are you currently not employing AI/ML based algorithms for RWD analysis?

24 Do you have access to external RWD sets?

- ( ) Yes
- ( ) No
- ( ) I don't know

25 What types of RWD sets do you have access to?

- [ ] Electronic health records (EHRs)
- [ ] Disease or patient registries
- [ ] Medical claims and billing data
- [ ] Prescription records (pharmacy dispensing data)
- [ ] Patient-generated data (e.g. from wearables or apps)
- [ ] Social media based data
- [ ] Patient-powered research networks
- [ ] Laboratory results (e.g. imaging data)
- [ ] Biobanks or genetic databases
- [ ] Other
- [ ] I don't know

26 Which other types of external RWD sets do you have access to?

27 Which RWD sources would you like to use, but currently cannot?

- [ ] Electronic health records (EHRs)
- [ ] Disease or patient registries
- [ ] Medical claims and billing data
- [ ] Prescription records (pharmacy dispensing data)
- [ ] Patient-generated data (e.g. from wearables or apps)
- [ ] Social media based data
- [ ] Patient-powered research networks
- [ ] Laboratory results (e.g. imaging data)
- [ ] Biobanks or genetic databases
- [ ] Other
- [ ] I don't know

28 Which other types of RWD sources would you like to use, but currently cannot?

29 Do you already make use of a common data model (CDM) when working with RWD?

- ( ) Yes
- ( ) No
- ( ) I don't know

30 Which CDM do you use when working with RWD?

- [ ] PCORnet
- [ ] EU-ADR
- [ ] FDA Sentinel
- [ ] IMI-PROTECT
- [ ] i2b2
- [ ] CNODES
- [ ] OMOP
- [ ] Other
- [ ] Sentinel CIDA
- [ ] I don't know
- [ ] Aetion

31 Which other CDM do you use when working with RWD?

Interest in RWD/RWE and AI/ML

32 Are you interested in (AI/ML-based) tools for the analysis of RWD/RWE?

- ( ) Yes
- ( ) No

33 In (AI/ML-based) tools for which applications/use cases are you particularly interested in?

34 Are you interested in participating in training sessions or educational programs to learn more about the use RWD /RWE?

- Yes
- No

35 In what format would you prefer the educational programs to take place?

Use drag&drop or the up/down buttons to change the order or accept the initial order.

- (Self-paced) online courses
- Online training (seminars, workshops)
- In-person training (seminars, workshops)
- Video tutorials
- Blended learning: Combination of in-person events, online events, and self-learning

36 Are you interested in good practice examples related to the use of RWD/RWE for regulatory affairs and/or HTA purposes?

- Yes
- No

37 You are interested in good practice examples using RWD/RWE. For which purposes specifically?

- Pre-authorization efficacy assessment
- Post-authorization efficacy/effectiveness analysis
- Pre-authorization safety assessment
- Post-authorization safety analysis
- Contextualization: Analysis of disease epidemiology, disease progression, and response to available treatments
- Assessment of unmet medical need
- Selection of patients for clinical trials
- Indirect treatment comparison
- Selection of endpoints for clinical trials
- Other
- Single-arm trials using historic/external control groups
- I don't know
- Target trial emulation

38 For which *other* purposes are you specifically interested in good practice examples using RWD/RWE?

End

39 We value your insights and experiences in the realm of Real-world data (RWD) and Real-world evidence (RWE) implementation. In this open-ended question, we invite you to share your perspectives on two key aspects:

- Obstacles and challenges: What, in your opinion, are the most significant challenges or obstacles that currently hinder the effective implementation of RWD and RWE in drug regulatory decision-making and Health Technology Assessment (HTA)? Please feel free to elaborate on any technical, organizational, regulatory, or other types of barriers you have encountered or anticipate.

- Additional remarks: Is there anything else you would like to share with us regarding the use of RWD and RWE? This could include suggestions for improvement, areas needing more attention, or any other comments you believe are pertinent to our project's objectives.

Your input is invaluable in guiding the development of standards, methods, and training concepts for the use of RWD and RWE. Thank you for taking the time to contribute your expertise and insights.

Thank you

Thank you very much for completing this survey. Your input is greatly appreciated.

Please click the **submit** button to submit your answers.

You can stay up-to-date on Real4Reg by following the links on the sidebar.

Real4Reg

Real4Reg Survey - Patients and Physicians

Introduction

Welcome to the Real4Reg Survey on Real-World Data and Real-World Evidence use in Regulatory Affairs and Health Technology Assessment

Background

Real-world data (RWD) and real-world evidence (RWE) are increasingly recognized as important tools in healthcare. In simple terms, RWD refers to health data routinely collected during patient interactions within healthcare settings. This includes, among other things, data from various sources such as doctor or hospital visits, pharmacy prescription records, and patient registries.

This data has huge amounts of valuable information hidden within it: Information about how drugs work in real world settings, how effective they are, who benefits from them and who doesn't, information about how drugs interact with each other, and so on. Generally, RWD/RWE has huge potential for advancing medical research. This information obtained by analysing RWD is called real-world evidence (RWE).

RWD is particularly promising in clinical drug research. Clinical research refers to studies where drugs get tested for safety and efficacy in humans. As of now, most clinical research is conducted in the form of randomized clinical trials (RCTs). While having important advantages on their own, RCTs are limited in other aspects. They are an artificial setting, which doesn't represent the real world 1:1. This means drugs might behave differently in the real world than in the RCT. For example, RCTs only recruit people with a very specific set of characteristics (e.g. people with comorbidities are often excluded, pregnant women and children are excluded); this group of people differ from people using the drugs in the real world, i.e., potentially everyone, including children and teenagers, elderly, and (pregnant) women. Using RWD/RWE can help to supplement RCTs in these aspects and thus improve the quality of drugs available to patients.

The Real4Reg Project

The Real4Reg project is a European public research initiative, consisting of European drug regulatory agencies, universities, and patient organisations, with the shared goal of promoting the use of RWD and RWE in medical and drug research. For an in-depth look at our work, visit our website at https://www.real4reg.eu/.

Aim of this survey

To make sure RWD/RWE will be used in a way where everyone profits, we want to understand the current state of knowledge people have of the topic of RWD/RWE, find out people's opinions, and identify concerns they have. Ultimately, patients and physicians like you are at the very core of the healthcare system; thus

we consider it very important to take your perspective into account. Receiving feedback and information from patients and physicians is crucial for the acceptance of RWD/RWE-based research and we hope you can help us to improve clinical drug research by telling us your opinion on this matter.

How to complete the survey

Answering the survey will take approximately 10-15 minutes. When answering questions in this survey, please make sure to click the small question mark (?) symbol next to some questions.

By participating in this survey, you are helping us to develop a future where RWD is used in a responsible way where everyone profits.

Thank you for participating!

Data protection

Data collection within this survey

Your data is collected for the purpose of scientifically analysing the current state of Real-World Data (RWD) and Real-World Evidence (RWE) usage, expertise, and the stakeholder's general opinions towards this topic.

All data collected as part of this survey is anonymized. This means, your responses will not be identifiable to you as an individual. All data gathered during this survey will be aggregated and analysed. In order to stay anonymous, please do not insert any personal information into the freetext fields in this survey. We will delete any personal information you might insert into the free text fields to keep the survey anonymous.

The results of this analysis will be used to steer the development of training materials, and the general communication and dissemination strategy in the Real4Reg project with the goal of facilitating the adoption and implementation of RWD and RWD usage in healthcare decision-making in Europe. The anonymized results of th survey will be published in relevant contexts, e.g., on the Real4Reg website or in a peer-reviewed journal. No further processing of the data for any other purposes outside the scope of this specific context is intended.

Data collection method

For the collection of data in this survey, the external EU survey system "EUSurvey" will be used. For more information on how EUSurvey processes personal data, please see: https://ec.europa.eu/eusurvey/home /privacystatement

This survey will use the "Anonymous survey mode", which means that EUSurvey will not save any personal data such as IP addresses.

Contact information

Publisher and responsible for this survey is the Bundesinstitut für Arzneimittel und Medizinprodukte (BfArM, Federal Institute for Drugs and Medical Devices), more specifically the research group "Pharmakoepidemiologie" of the Research Department.

Address: Kurt-Georg-Kiesinger-Allee 3, 53175 Bonn, Germany
E-Mail: Real4Reg@bfarm.de

General Information

1 In which country do you live?

- Austria
- Belgium
- Bulgaria
- Croatia
- Cyprus
- Czechia
- Denmark
- Estonia
- Finland
- France
- Germany
- Greece
- Hungary
- Ireland
- Italy
- Latvia
- Lithuania
- Luxembourg
- Malta
- Netherlands
- Poland
- Portugal
- Romania
- Slovak Republic
- Slovenia
- Spain
- Sweden
- United Kingdom
- Switzerland
- Other

2 In which other country do you live?

3 Are you answering this survey as a patient or in your role as a physician?

- Patient
- Physician

4 What is your age?

- 18-29
- 30-39
- 40-49

- 50-59
- 60-69
- 70+

5 Please indicate whether you have a health condition that falls into one of the following categories:

- Chronic disease
- Rare/orphan disease
- Both chronic and rare/orphan disease
- Neither
- I don't know / I wish not to share this information

6 Which best describes the primary setting of your medical practice?

- Hospital with a dedicated research component (e.g. university hospital)
- Care-oriented hospital without significant research activities (e.g. "normal" hospital)
- Private practice
- Hybrid practice
- Other

7 Are you a member of a patient organization, a medical association, or a similar society?

- Yes
- No
- I don't know

8 Are you registered in a patient/disease registry?

- Yes
- No
- I don't know

9 Are you sharing your health data in other way for research purposes?

- Yes
- No
- I don't know

10 In what other way are you sharing your health data for research purposes?

Knowledge about RWD/RWE and AI/ML

11 Have you heard about RWD/RWE in the context of healthcare data before?

RWD: real-world data
RWE: real-world evidence

- Yes
- No
- I don't know

12 Were you ever educated (e.g. by your physician) about the topic of RWD usage for medical research purposes?

- Yes
- No
- I don't know

13 Have you ever received any vocational training regarding the topic of RWD usage for medical research purposes?

- Yes, I have received formal vocational training on this topic (e.g. in the setting of continuing medical education (CME))
- Yes, I have received training on this topic, but not in a formal setting (e.g. in voluntary seminars or conferences).
- Yes, but only short informational briefs.
- No

14 Do you feel sufficiently knowledgable and confident to educate your patients on the topic of using RWD/RWE for medical research purposes?

- Completely confident
- Somewhat confident
- Not very confident
- Not confident at all

15 Please rate your current level of proficiency for each RWD related topic

RWD: real-world data

RWE: real-world evidence

AI: artificial intelligence

ML: machine learning

No knowledge: Completely unfamiliar with the topic.

Minimal knowledge: Recognize basic terms or concepts, but not how they apply or function.

Basic knowledge: Understanding of fundamental concepts, can handle simple tasks, but limited hands-on experience and no deeper understanding.

Intermediate knowledge: Good understanding with practical experience, confident in common situations.

Advanced knowledge: Deep understanding with significant experience, can navigate complex situations, is sought for advice on the topic by others.

Expert knowledge: Authority in the topic, extensive experience; can teach, mentor and lead in this topic.

	No knowledge	Minimal knowledge	Basic knowledge	Intermediate knowledge	Advanced knowledge	Expert knowledge
**Clinical research & drug development basics:** Basics of the clinical drug development process; how drugs get tested for efficacy and safety; key aspects of clinical trials; basics of the regulatory process.	◯	◯	◯	◯	◯	◯

**Real-world data basics: What is RWD?** Types and sources of RWD; the role of RWD in medical research.	○	○	○	○	○	○
**Applications of RWD in clinical drug development:** How RWD/RWE can be applied to clinical trials to make drug development safer and faster; how RWD can be used to include underrepresented populations (e.g. pregnant women, children, people with comorbidities); how RWD can facilitate clinical research on rare diseases.	○	○	○	○	○	○

**AI/ML-based algorithms and RWD:** Basic principles of AI /ML technology; how AI/ML-based algorithms accelerate RWD-based research; safety and limitations of AI /ML-based algorithms.	○	○	○	○	○	○
**Data privacy and ethics in RWD usage:** Understanding data privacy laws and regulations; patient rights and protections; ethical principles of RWD usage and how they are guaranteed.	○	○	○	○	○	○

**Patient centric approaches to drug development and individualized medicine:** The role of patients and physicians in drug development; the importance of patient advocacy and feedback; implications of RWD for individualized medicine.	○	○	○	○	○	○

16 Please indicate how important/useful you think knowledge about RWD/RWE related topics will be for you in the future.

RWD: real-world data
RWE: real-world evidence
AI: artificial intelligence
ML: machine learning

Not important: See no relevance or necessity in future contexts.
Slightly important: Recognize some potential relevance, but uncertain of its practical importance.
Moderately important: Potentially useful in some scenarios, but not a general requirement.
Important: View it as an important topic.
Very important: Foresee it becoming central to many aspects of healthcare; crucial for informed decision-making.
Absolutely essential: Will be indispensable knowledge in the future.

	Not important	Slightly important	Moderately important	Important	Very important	Absolutely essential
**Clinical research & drug development basics:** Basics of the clinical drug development process; how drugs get tested for efficacy and safety; key aspects of clinical trials; basics of the regulatory process.	○	○	○	○	○	○
**Real-world data basics: What is RWD?** Types and sources of RWD; the role of RWD in medical research.	○	○	○	○	○	○

**Applications of RWD in clinical drug development:** How RWD/RWE can be applied to clinical trials to make drug development safer and faster; how RWD can be used to include underrepresented populations (e.g. pregnant women, children, people with comorbidities); how RWD can facilitate clinical research on rare diseases.	○	○	○	○	○	○
**AI/ML-based algorithms and RWD:** Basic principles of AI/ML technology; how AI /ML-based algorithms accelerate RWD-based research; safety and limitations of AI/ML-based algorithms.	○	○	○	○	○	○

**Data privacy and ethics in RWD usage:** Understanding data privacy laws and regulations; patient rights and protections; ethical principles of RWD usage and how they are guaranteed.	○	○	○	○	○	○
**Patient centric approaches to drug development and individualized medicine:** The role of patients and physicians in drug development; the importance of patient advocacy and feedback; implications of RWD for individualized medicine.	○	○	○	○	○	○

17 In general, how significant do you think will be the impact of RWD/RWE for patient care in the future?

- Not important at all
- Slightly important
- Moderately important
- Important
- Very important
- Absolutely essential

18 How important do you believe is it to educate patients and physicians about RWD/RWE and how it will affect patients and impact healthcare outcomes?

- Not important at all
- Slightly important
- Moderately important
- Important
- Very important
- Absolutely essential

19 "The current healthcare system sufficiently takes into account the opinions and experiences of patients regardings drugs and other treatments."
Do you agree with this statement?

- I strongly agree
- I somewhat agree
- I somewhat disagree
- I strongly disagree
- I don't know

20 For what purposes would you be willing to share your personal health-related data?

- **Academic research:** Allow researchers at universities and public research institutes data access to support basic research.
- **Private sector research:** Enable pharmaceutical companies to use RWD for clinical drug research & development and to supplement clinical studies.
- **Regulatory and other government agencies:** Allow agencies like drug regulatory agencies data access in order to improve the evaluation mechanisms for drugs to better evaluate the safety and efficacy of treatments.
- **Education and professional training:** For educational purposes in medical institutions.
- **Public health research & monitoring:** To support public health studies and governmental health planning.
- **Not willing to share for any purpose**

21 What are your expectations regarding how RWD/RWE usage for clinical research will contribute to better management of your personal/your patient's health condition?

- **Improved diagnostic accuracy and speed**
- **Personalized treatment plans:** Treatment plans better taking the individual characteristics of each patient into account.
- **Improved drug development:** More effective and safer medications.
- **Improved drug availability**: Access to a wider range of drugs and other treatment options.
- **Better disease monitoring:** Improved management of chronic diseases.

- [ ] **Increased patient engagement:** Greater participation of patients in their own healthcare decision-making and improved consideration of patient feedback for drug development.
- [ ] **Better healthcare availability and delivery:** Reduced waiting times and better acessibility of the healthcare system.
- [ ] **Reduction of healthcare costs**
- [ ] **I do not expect any benefits for patients**
- [ ] **Other**
- [ ] **I don't know**

22 What other positive outcomes for patients do you expect from RWD/RWE usage for clinical research?

23 What are your main concerns regarding the use of RWD/RWE for clinical research purposes?
- [ ] **Privacy, security, and ethical issues:** Concerns about the sufficient scope of data privacy legislation, as well as potential breaches or other misuse of patient data.
- [ ] **Interpretation and application in decision-making:** Risk of misinterpretation of RWD leading to worse healthcare outcomes.
- [ ] **Infrastructure and technology challenges:** Difficulties regarding the implementation of the technological infrastructure necessary for collecting and using RWD in a reliable way.
- [ ] **Impact on patient care and treatment outcomes:** Doubts whether the concerns and needs of patients will be properly taken into account and whether RWD/RWE usage will truly lead to positive outcomes for patients.
- [ ] **Professional training and understanding:** Ability of the medical community to incorporate RWD/RWE into daily practice, to best advice patients on this topic.
- [ ] **Workload and administrative burden:** Concerns about the potential increase in workload and administrative tasks for physicians, stemming from the integration of RWD/RWE into regular practice.
- [ ] **I do not have any concerns**
- [ ] **Other concerns**
- [ ] **I don't know**

24 What other concerns or reservations regarding the use of RWD/RWE for research purposes do you have?

Interest in RWD/RWE

25 Are you interested in participating in training sessions or educational programs to learn more about the use of RWD/RWE for medical and clinical research, including its implications for patients and physicians?
- ○ Yes
- ○ No

26 In what format would you prefer the educational programs to take place?
Use drag&drop or the up/down buttons to change the order or accept the initial order.

- (Self-paced) online courses
- In-person training (seminars, workshops)

- Online training (seminars, workshops)
- Video tutorials
- Blended learning: Combination of in-person events, online events, and self-learning

27 What source do you typically use to stay informed about new developments related to your health condition?

- [ ] Physicians and other healthcare providers
- [ ] Medical news websites for lay audiences
- [ ] Medical news websites for professional audiences
- [ ] Other online health portals
- [ ] Social media and online forums
- [ ] Support groups
- [ ] Newsletters
- [ ] Print media
- [ ] Other

28 What other sources do you typically use to stay informed about new developments related to your health condition?

29 How do you usually keep up-to-date with the latest developments in your medical field?

- [ ] Medical journals and scientific publications
- [ ] Professional conferences and seminars
- [ ] Continuing medical education (CME) or other formal training frameworks
- [ ] Peer networks
- [ ] Research collaborations, disease associations, or other professional networks
- [ ] Newspapers for professional audiences
- [ ] Other

30 Via what other means do you usually keep up-to-date with the latest developments in your medical field?

End

31 In this open-ended question, we invite you to share your perspectives on two key aspects:

- Obstacles and challenges: As a patient or physician, what do you perceive as the most significant challenges or obstacles in effectively utilizing RWD and RWE for research purposes and in the healthcare system overall. Please feel free to discuss any personal experiences, practical difficulties, understanding gaps, and other concern you have.
- Additional remarks: Is there anything else you would like to share with us regarding the use of RWD and RWE? This could include suggestions for how these data could be better used in patient care, areas that needs more focus, or any other thoughts.

Your input is invaluable in guiding the development of standards, methods, and training concepts for the application of RWD and RWE for medical research and clinical drug development purposes; and especially for making sure that the opinions and experiences of patients (and physicians) are respected and taken into account. Thank you for taking the time to contribute your expertise and insights.

Thank you

Thank you very much for completing this survey. Your input is greatly appreciated.

Please click the **submit** button to submit your answers.

You can stay up-to-date on Real4Reg by following the links on the sidebar.
