# Supplementary material for: Key Stakeholders' Knowledge, Opinions, and Interests on Real‐World Evidence in the Regulatory Process—Results of an EU‐Wide Survey
Source: Clin Transl Sci. 2025 Dec 20;18(12):e70454. doi: 10.1111/cts.70454 (PMC12717853; doi:10.1111/cts.70454)
Supplement: Supplementary file 1 — Data S1: Supporting Information. [file CTS-18-e70454-s001.zip › CTS-2025-0743-T-s02.docx]

| Country | Count |
| --- | --- |
| Austria | 2 |
| Belgium | 2 |
| Croatia | 1 |
| Denmark | 2 |
| Finland | 2 |
| Germany | 84 |
| Ireland | 3 |
| Italy | 3 |
| Netherlands | 5 |
| Norway | 1 |
| Poland | 1 |
| Portugal | 5 |
| Romania | 1 |
| Spain | 3 |
| Switzerland | 4 |
| United Kingdom | 6 |
| Not specified | 1 |
| Total | 126 |

Table S1: Countries of origin for participants in survey I

| Country | Count |
| --- | --- |
| Austria | 1 |
| Czechia | 1 |
| Denmark | 2 |
| Finland | 2 |
| France | 1 |
| Germany | 9 |
| Greece | 1 |
| Italy | 3 |
| Netherlands | 4 |
| Portugal | 1 |
| Spain | 2 |
| Sweden | 1 |
| Ukraine | 1 |
| United Kingdom | 5 |
| United States | 1 |
| Not specified | 1 |
| Total | 36 |

Table S2: Countries of origin for participants in survey II

| Country | Count |
| --- | --- |
| Belgium | 1 |
| France | 1 |
| Germany | 5 |
| Greece | 2 |
| Hungary | 1 |
| Italy | 1 |
| Netherlands | 2 |
| New Zealand | 1 |
| Norway | 1 |
| Portugal | 25 |
| Slovak Republic | 1 |
| Slovenia | 1 |
| South Korea | 2 |
| Spain | 1 |
| Tunisia | 1 |
| Turkey | 1 |
| Ukraine | 1 |
| United Kingdom | 2 |
| Not specified | 3 |
| Total | 53 |

Table S3: Countries of origin for participants in survey IV


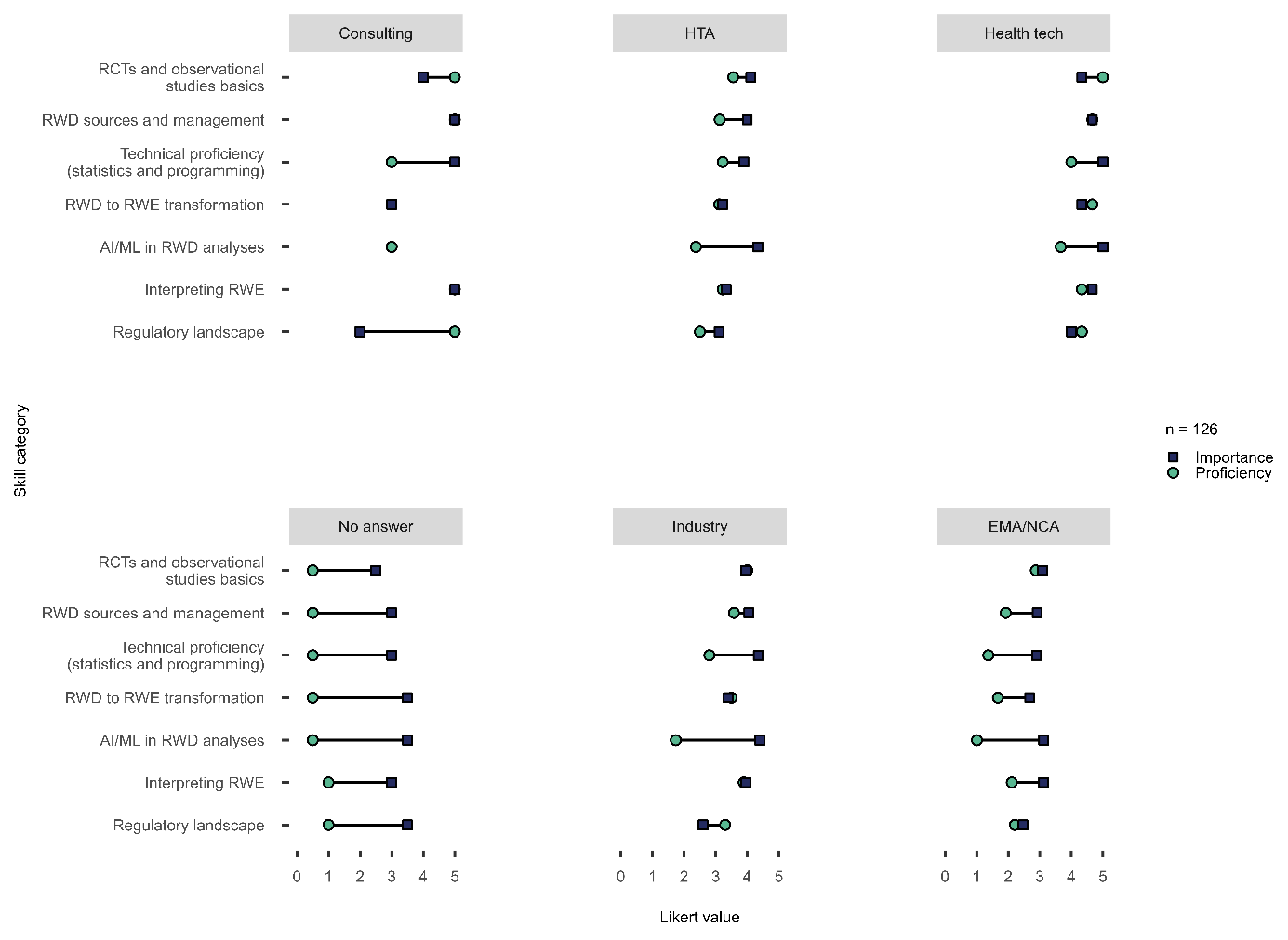


Figure S1: Average Likert scores of self-rated proficiency and perceived importance of different RWD-related topics for respondents of survey I (n=126), stratified by employer. Two respondents for Consulting, 9 for HTA, 3 for Health technology, 2 for No answer, 20 for Industry and 91 for EMA/NCA.


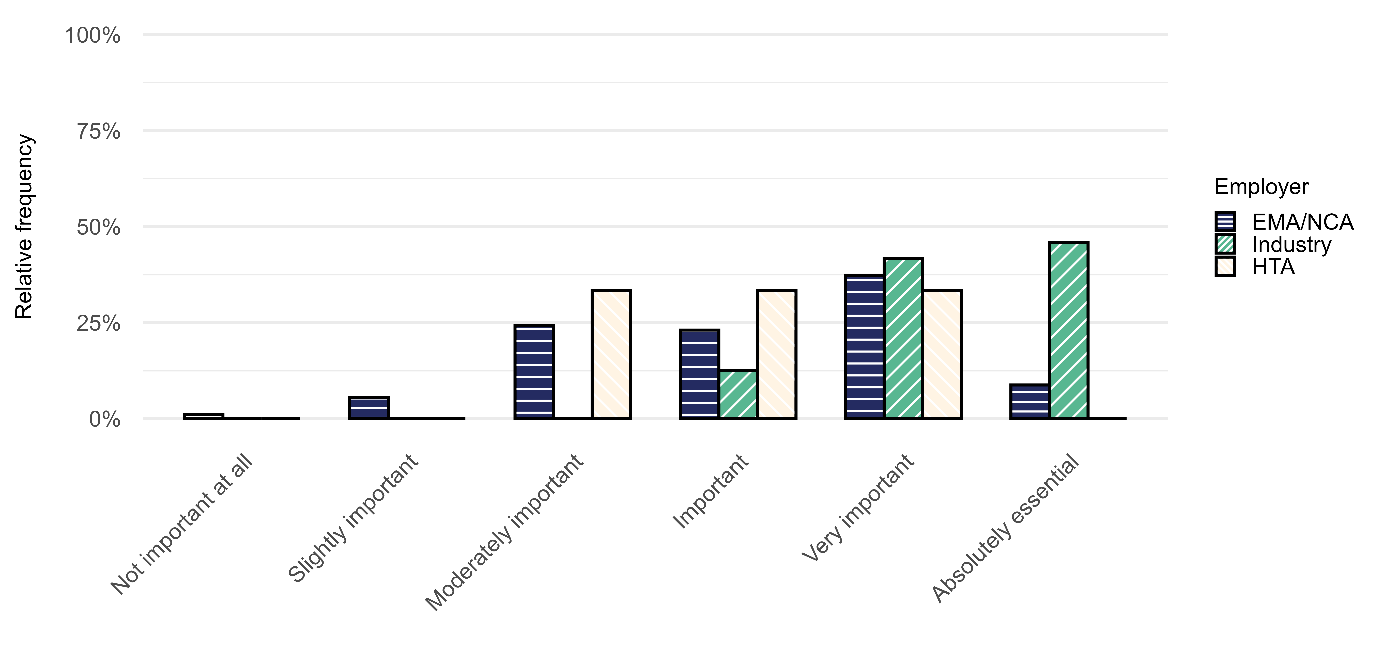


Figure S2: Survey I respondents’ rating of the future importance of RWD in regulatory decision-making, stratified by employer type. 91 respondents for EMA/NCA, 24 for Industry and 9 for HTA. Note that employer types Consulting, CRO and Health technology have been included in the Industry block.


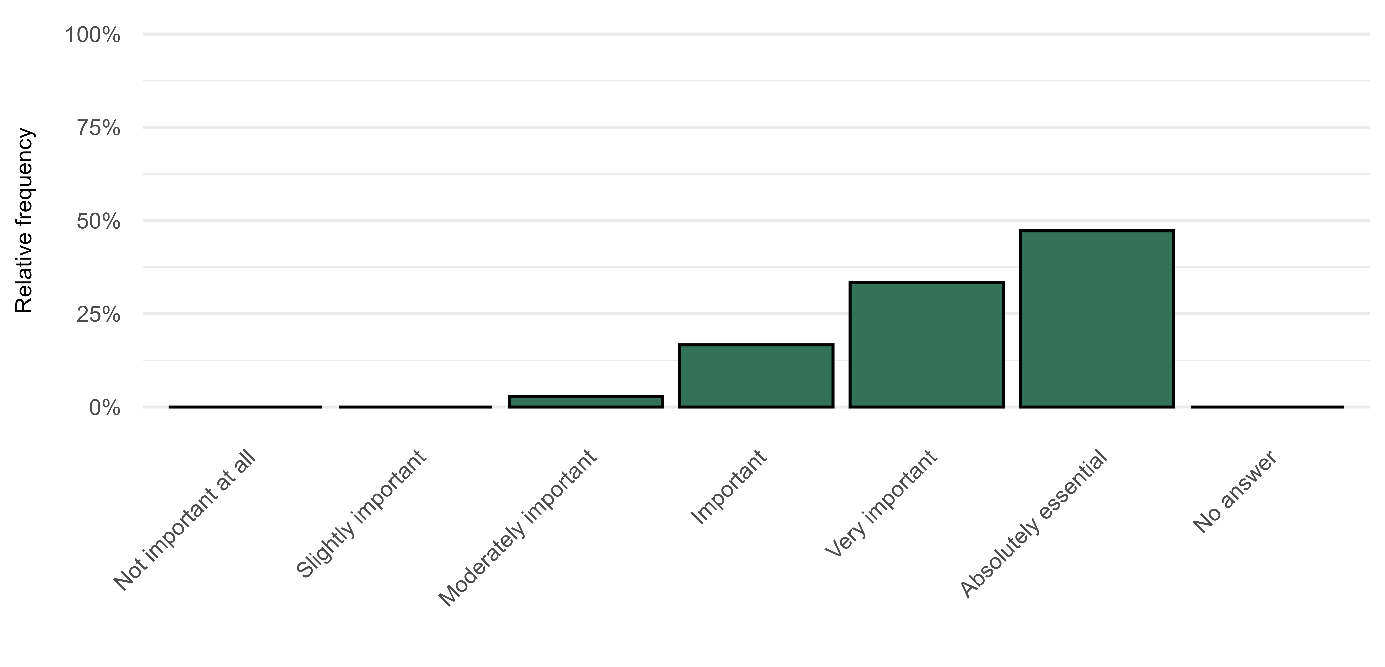


Figure S3: Survey II respondents’ rating of the future importance of RWD in regulatory decision-making (n = 36)


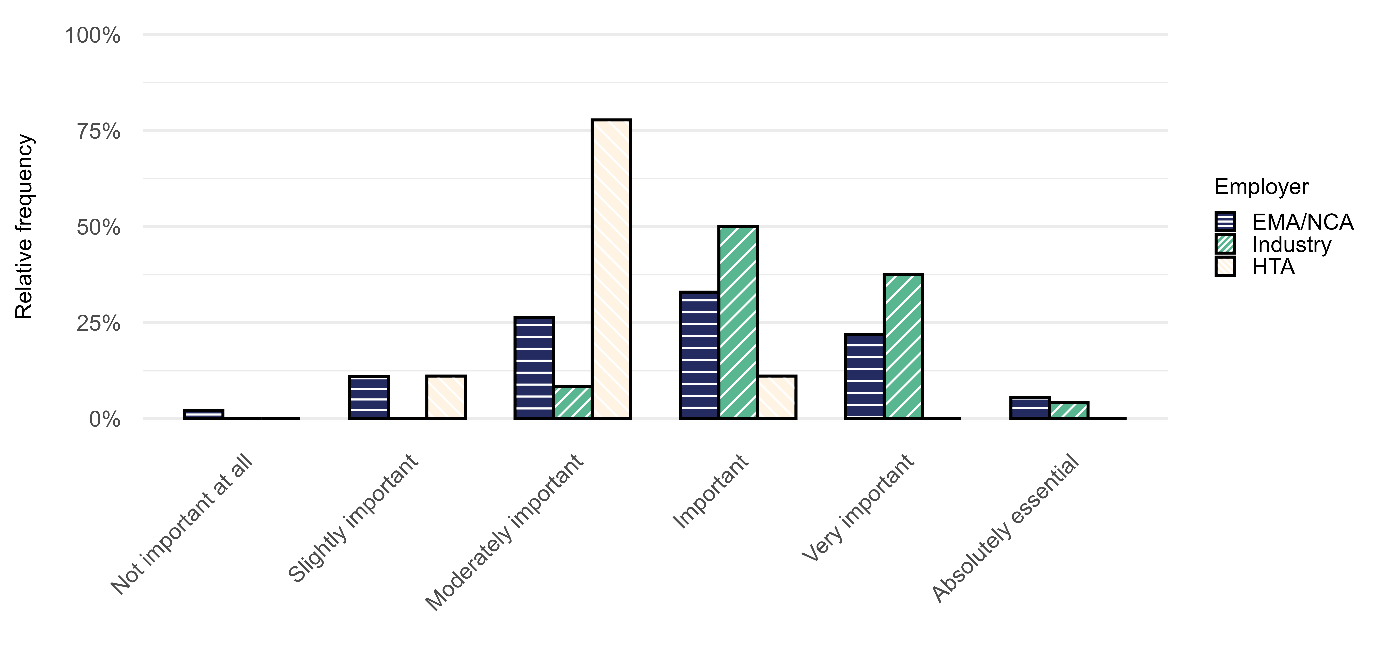


Figure S4: Survey I respondents’ rating of the future importance of AI/ML in regulatory decision-making, stratified by employer type. Note that employer types Consulting, CRO and Health technology have been included in the Industry block. 91 respondents for EMA/NCA, 24 for Industry and 9 for HTA.


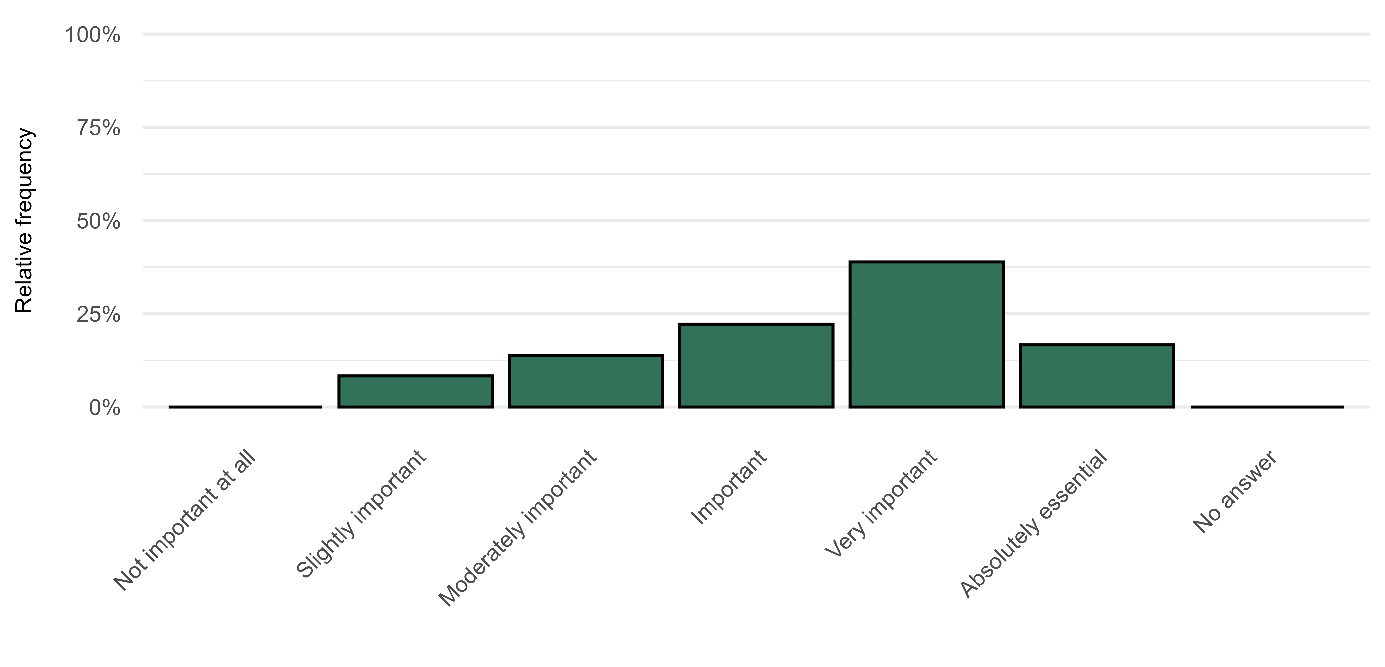


Figure S5: Survey II respondents’ rating of the future importance of AI/ML in regulatory decision-making (n = 36)


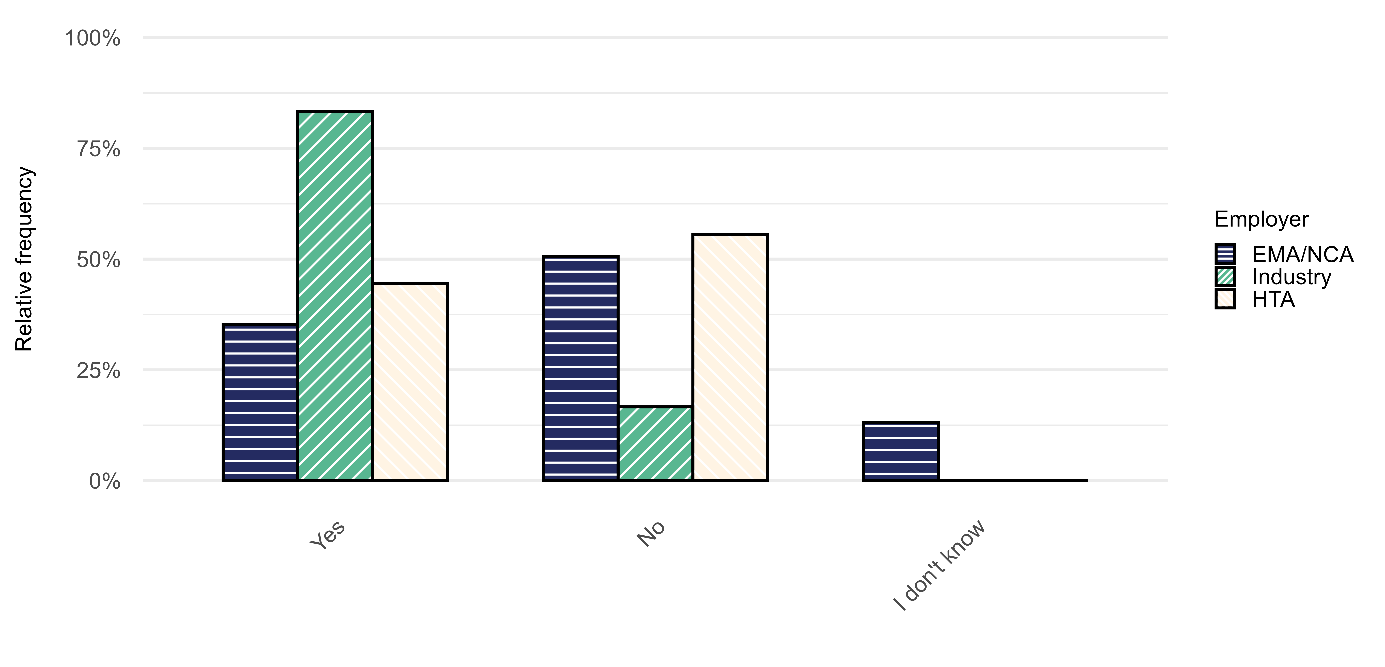


Figure S6: Survey I respondents' current use of RWD for regulatory purposes, stratified by employer type. Note that employer types Consulting, CRO and Health technology have been included in the Industry block. 91 respondents for EMA/NCA, 24 for Industry and 9 for HT
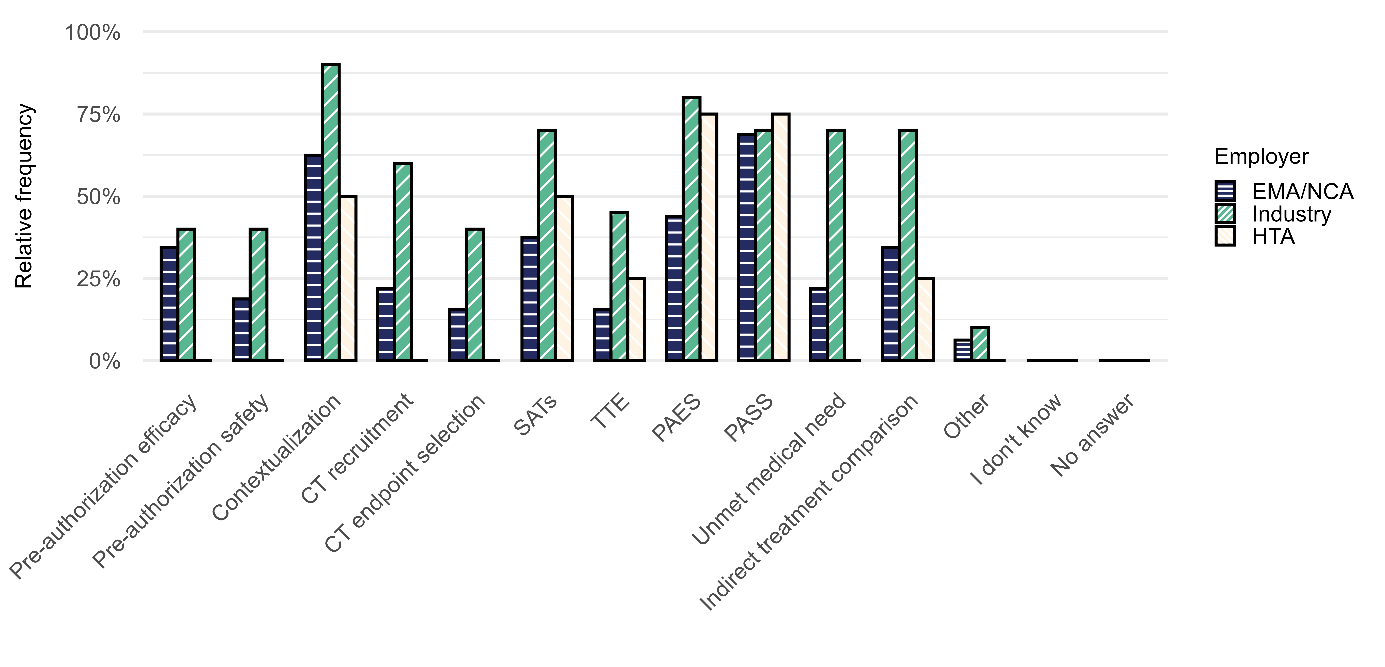
Figure S7: Current use cases of RWD for RWD users in survey I, stratified by employer type. Note that employer types Consulting, CRO and Health technology have been included in the Industry block. 32 respondents from NCA/EMA, 20 from Industry, 4 from HTA. More than one response could be selected.


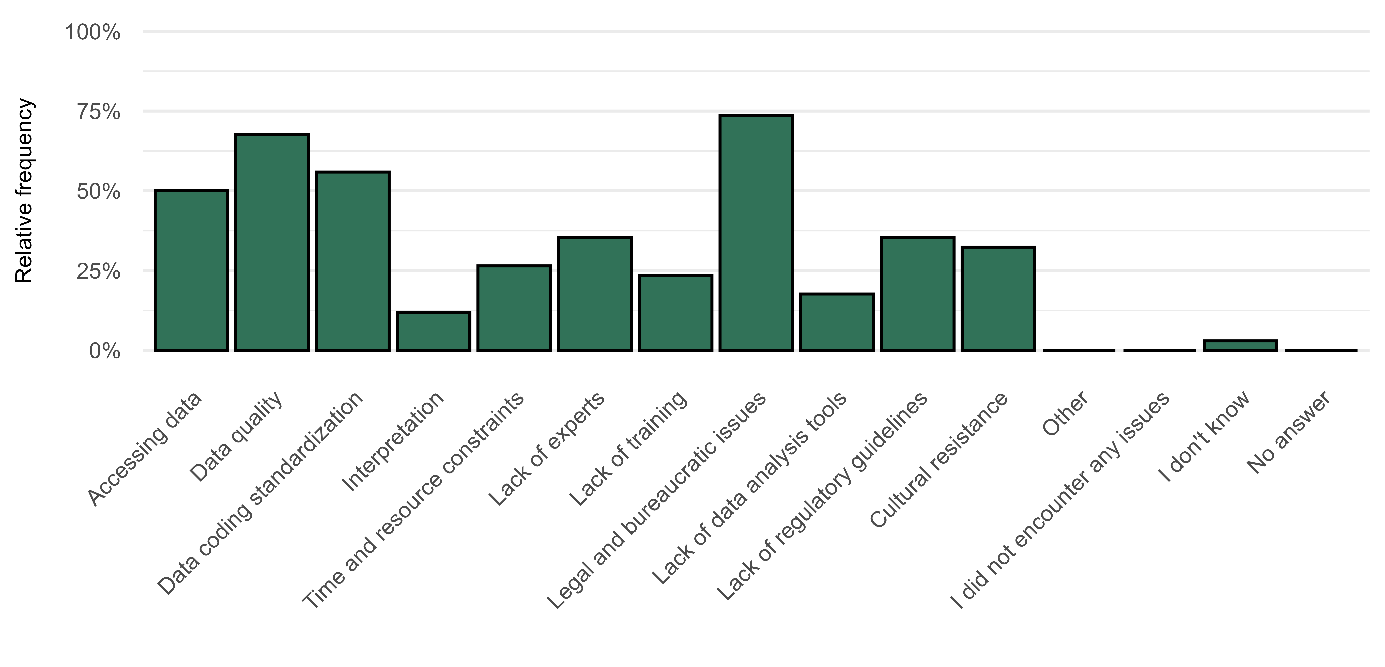


Figure S8: Challenges encountered when making use of RWD/RWE by RWD users in survey II (n = 32). More than one response could be selected.


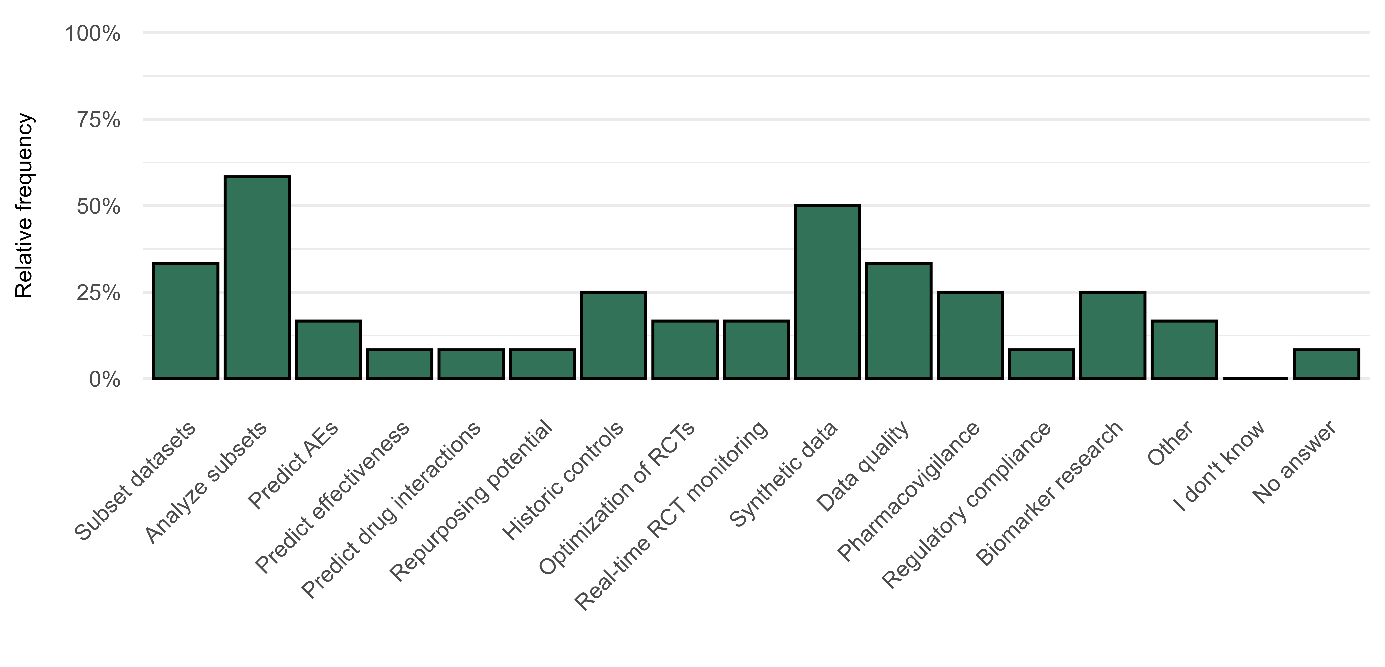


Figure S9: Current use cases of AI in RWD analyses for AI users in survey I (n = 12). More than one response could be selected.


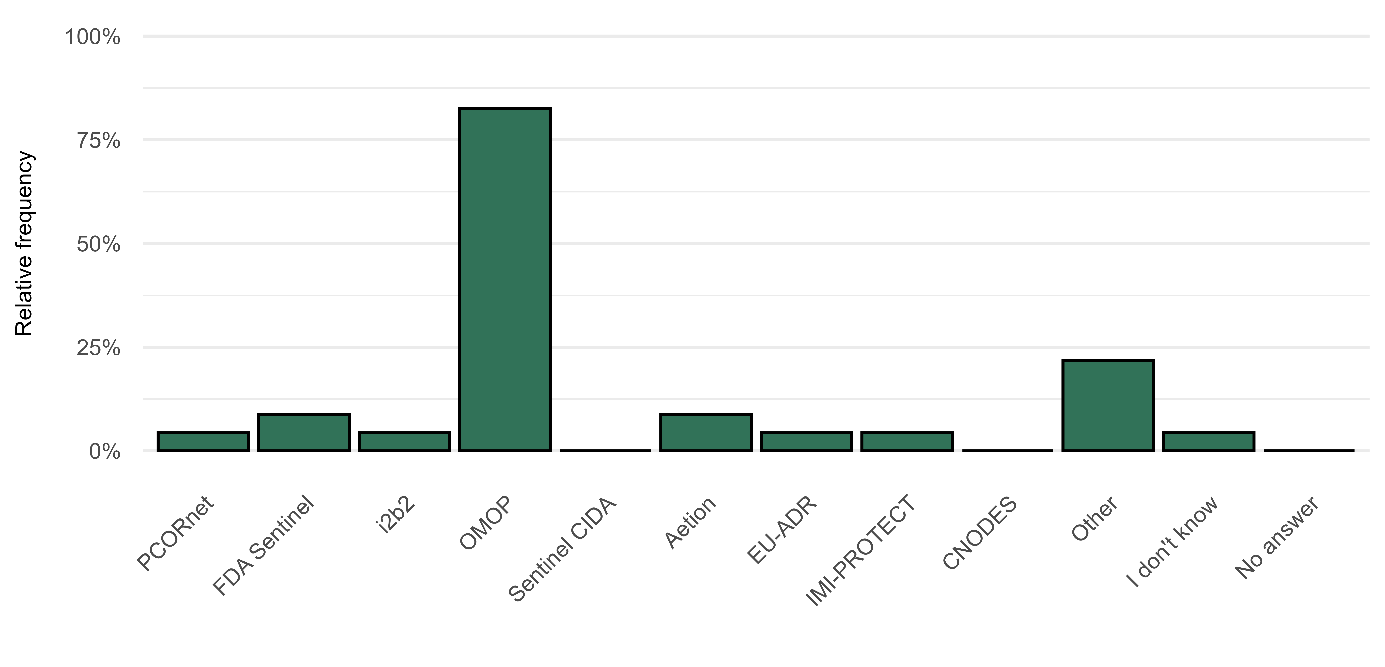


Figure S10: CDMs used by CDM users from survey I (n = 23). More than one response could be selected.


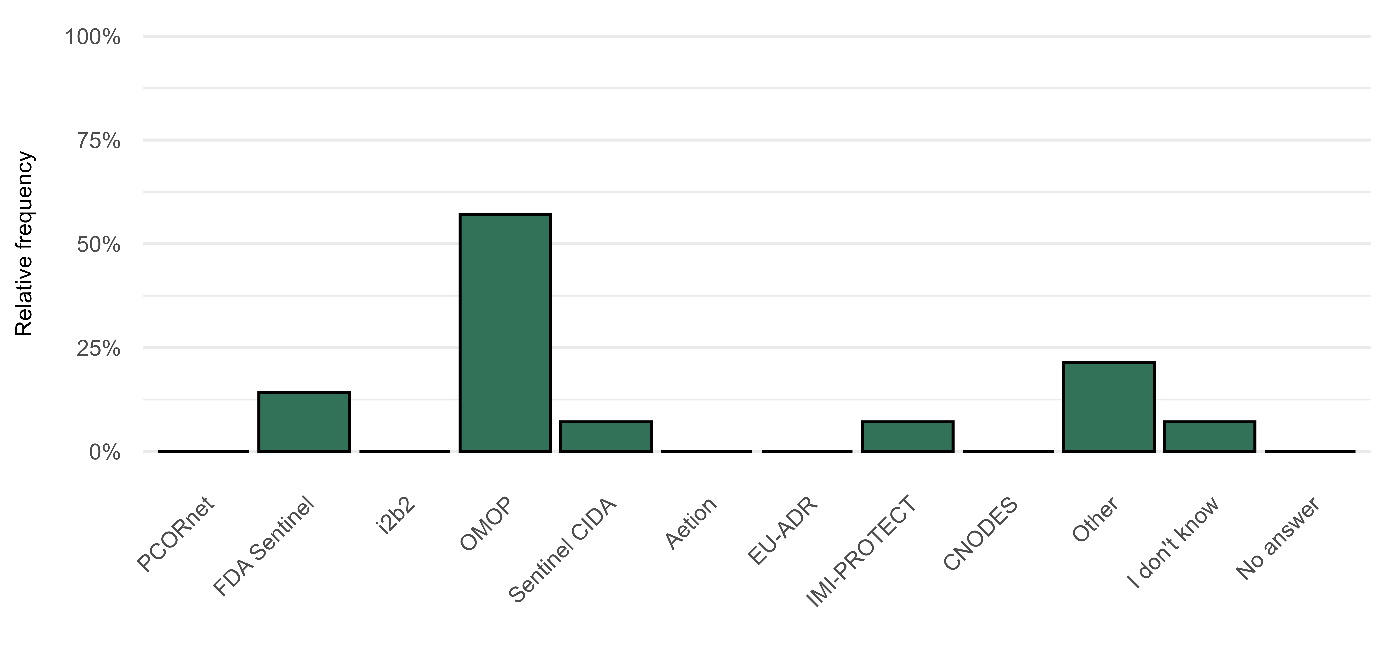


Figure S11: CDMs used by CDM users from survey II (n = 14). More than one response could be selected.


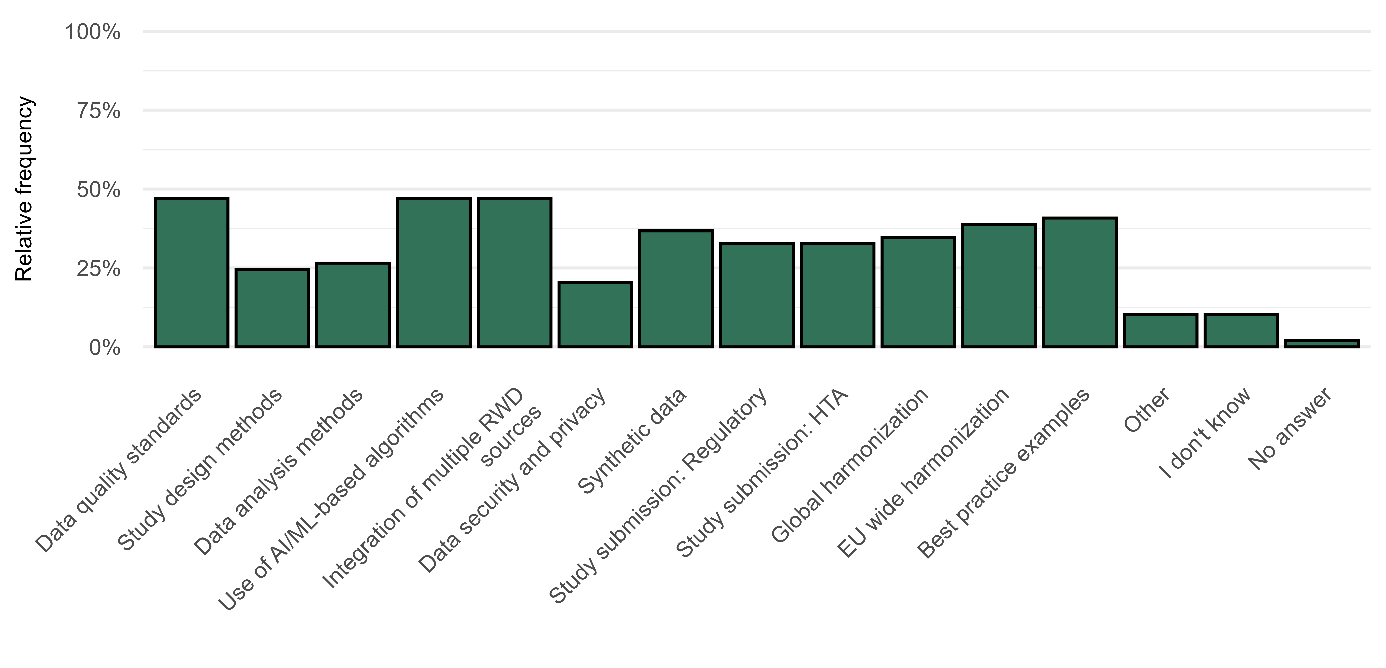


Figure S12: Gaps in current guidance identified by guidance users in survey I (n = 49). More than one response could be selected.


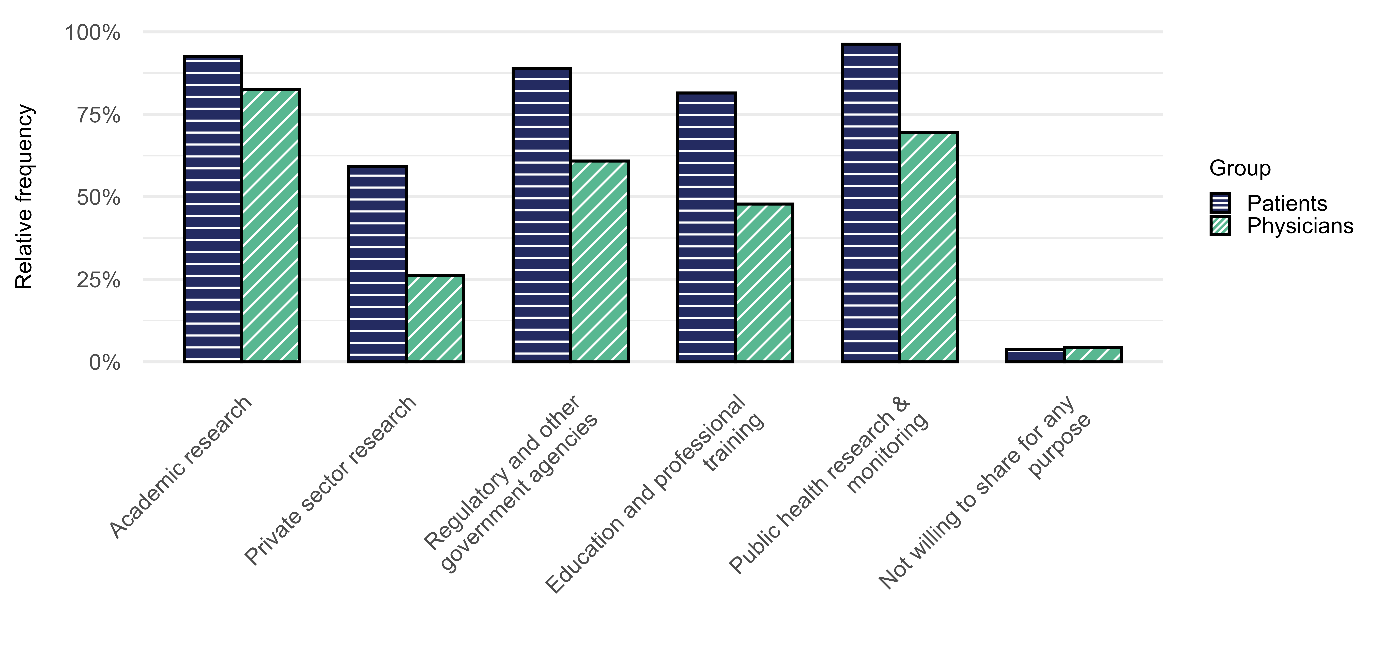


Figure S13: Willingness to share their healthcare data by respondent group in survey IV (n = 53). 27 respondents for Patients, 23 for Physicians. More than one response could be selected.
